# Fabrication of polydimethylsiloxane nanofluidic chips under AFM tip-based nanomilling process

**DOI:** 10.1186/s11671-019-2962-6

**Published:** 2019-04-17

**Authors:** Jiqiang Wang, Yongda Yan, Yanquan Geng, Yang Gan, Zhuo Fang

**Affiliations:** 1 Key Laboratory of Micro-systems and Micro-structures Manufacturing of Ministry of Education, Harbin Institute of Technology, Harbin, 150001 Heilongjiang People’s Republic of China; 2 Center for Precision Engineering, Harbin Institute of Technology, Harbin, 150001 Heilongjiang People’s Republic of China; 3 School of Chemistry and Chemical Engineering, Harbin Institute of Technology, Harbin, 150001 People’s Republic of China

**Keywords:** Atomic force microscopy, Nanomilling, Nanochannels, Nanofluidic chip

## Abstract

In current research realm, polydimethylsiloxane (PDMS)-based nanofluidic devices are widely used in medical, chemical, and biological applications. In the present paper, a novel nanomilling technique (consisting of an AFM system and a piezoelectric actuator) was proposed to fabricate nanochannels (with controllable sizes) on PDMS chips, and nanochannel size was controlled by the driving voltage and frequency inputted to the piezoelectric actuator. Moreover, microchannel and nanochannel molds were respectively fabricated by UV lithography and AFM tip-based nanomilling, and finally, PDMS slabs with micro/nanochannels were obtained by transfer process. The influences of PDMS weight ratio on nanochannel size were also investigated. The bonding process of microchannel and nanochannel slabs was conducted on a homemade alignment system consisted of an optical monocular microscope and precision stages. Furthermore, the effects of nanochannel size on electrical characteristics of KCl solution (concentration of 1 mM) were analyzed. Therefore, it can be concluded that PDMS nanofluidic devices with multiple nanochannels of sub-100-nm depth can be efficiently and economically fabricated by the proposed method.

**Electronic supplementary material:**

The online version of this article (10.1186/s11671-019-2962-6) contains supplementary material, which is available to authorized users.

## Background

Due to their considerable potentials in chemical, medical, and biological fields, micro/nanofluidic systems are widely used in DNA analysis [1–4], cell separation [5], protein research [6–8], food safety [9], and environmental monitoring [10]. With the rapid development of nanofabrication technology, the demand for nanofluidic devices with the one-dimensional size smaller than 100 nm is continuously increasing [11]. Nanofluidic chips can also be effectively used for virus detection [12], nanoparticle manipulation [13], and the study of ion diffusion [14]. However, the detection efficiency and sensitivity of the nanofluidic chips depend on the feature dimensions and distribution of the nanochannels. It is indispensable to accurately control the feature dimensions of the nanochannels for nanofluidic-based label-free detection. How to fabricate nanochannels with controllable feature dimensions and distribution is still a challenge for the application in the nanofluidic field.

Till now, there are several methods that can be utilized for the fabrication of nanofluidic chips. Reactive ion etching [15], conventional photolithography [16], high-energy beam processing [17], interference lithography [18], nanoimprinting [19], and hot embossing technologies [20, 21] are most commonly used for the fabrication of nanofluidic devices; however, all of these methods manifest their own limitations. Reactive ion etching and conventional photolithography are the mainstream methods for micro/nanofluidic channel fabrication. However, the lateral dimensions of the fabricated channels depend on the wavelength of the incident light, thus the widths of the produced channels are often found in the micrometer scale, not in nanoscale [22]. Besides, it is inconvenient to change the photomasks when fabricate micro/nanostructures have different features. Focused ion beam lithography (FIB) and electron beam lithography (EBL) both are high-energy beam processing methods, which can easily fabricate high-precision nanofluidic chip with sub-100 nm nanochannels. However, the investment for the fabrication facility is extremely high and the strict environmental requirement is necessary [23]. Interference lithography (IL) is suitable for fabricating simple periodical structures over a large area; however, it is not suitable to machine a single nanochannel [24, 25]. The processing resolution of nanoimprinting depends on template properties, the crucial issue for this approach is how to fabricate the template with high-precision nanostructures [26]. In addition, sacrificial molding and creak-based method are also adopted to fabricate micro/nanoscale devices [27, 28]; however, the accurate control of nanochannel size is very difficult in these approaches. Thus, a more feasible fabrication approach with the properties of high machining precision, ease of use, large processing range and low environment requirement is demanded for the fabrication of nanofluidic device.

In recent years, due to their high machining accuracies, ultra-precision machining methods, such as nanomilling, precision grinding, and ultra-precision turning, are widely used in micro/nanostructure fabrication [29–32]. Moreover, since the invention of atomic force microscope (AFM) in 1986, AFM tip-based nanofabrication is a powerful method to prepare nanostructures [33]. The traditional tip-based nanoscratching possesses some limitations, such as limited machining width and low fabrication efficiency. The width of the nanochannel fabricated by this approach is dependent on the geometry of the AFM tip, which signifies the nanochannels with controllable width which are inaccessible. In addition, the fabrication efficiency of the traditional tip-based nanoscratching process is relatively low especially for the case of employing a feed in the machining process to enlarge the depth and width of the obtained nanostructure. Due to its significant advantages, such as controllable machining size and high fabrication efficiency, tip-based nanomilling is widely adopted to fabricate nanochannels. Gozen et al. [34, 35] fabricated nanostructures on polymethyl methacrylate (PMMA) through a nanomilling process. Zhang et al. [36–38] prepared three-dimensional nanostructures using AFM and studied the effects of different machining parameters. Park et al. [39] investigated the mechanism of nanomachining process and found that the intensities of cutting force were significantly reduced; however, in the proposed system, the machining facilities were found to be relatively complicated and the material removal process was not investigated in details. The relationship between the machining parameters including the driving frequency and voltage and the feature dimensions of obtained nanochannel was not studied. In addition, their work did not focus on the application of the fabricated nanochannels. Therefore, more work is needed to explore the application scope of this AFM tip-based nanomilling approach. Polycarbonate (PC), due to its excellent machinability, is commonly used for nanofabrication [40]; nevertheless, it is rarely selected to fabricate nanofluidic chips. In contrast, polydimethylsiloxane (PDMS) is widely used to process microfluidic and nanofluidic chips. Mata et al. [41] studied the influences of PDMS weight ratio on tensile stress. Park et al. [42] developed a new method to improve the stiffness of PDMS. The applications of nanofluidic chips in label-free test field mainly depend on the electrical conductivity of nanochannels [43], thus the measurement results are often affected by the dimensional sizes of nanochannels [44].

Therefore, in order to overcome the disadvantages of traditional tip-based scratching process, the nanomilling approach is employed to conduct the fabrication process of nanochannel in this study. Moreover, PC sheet was selected as the experimental sample to mitigate tip wear as well as to reduce fabrication cost. Further, nanochannel size on PC sheet was controlled by the driving voltage and frequency inputted to the piezoelectric actuator. The influences of PDMS weight ratio on nanochannel size were also investigated. Furthermore, in order to verify the effects of different dimensional sizes on electric conductivity of nanochannels, the current measurement test was performed using KCl solution.

## Methods

### Nanomilling system setup

**Fig. 1:**
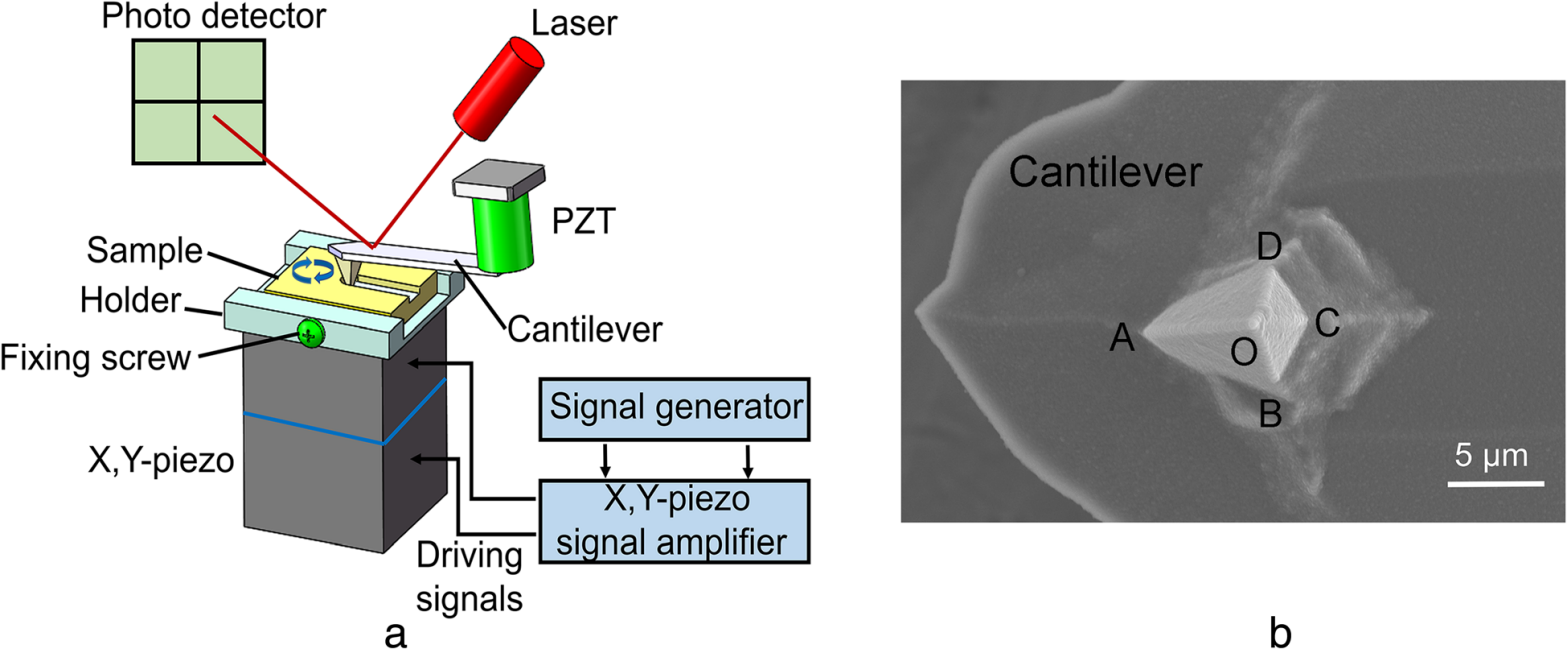
**a** Schematic diagram of nanomilling system. **b** SEM micrograph of diamond-coated AFM tip

The proposed AFM tip-based nanomilling system consisted of a commercial AFM (Dimension Icon, Bruker Company, Germany) and a piezoelectric actuator (P-122.01, PI Company, Germany) (Fig. 1a). The travel ranges of the piezoelectric actuator in both x- and y-directions were limited to 1 μm. Moreover, the piezoelectric actuator was driven by sinusoid signals with appropriate voltages (generated from a commercial signal generator device (AFG1022; Tektronix, Inc., USA)) under the amplification of a signal amplifier (PZD350A; TREK, Inc., USA). A PC sheet was fixed on the homemade holder (made of epoxy resin) by a fixing screw. The nanomachining operation was performed using a rectangular pyramidal diamond-coated tip of thickness 100 nm (DT-NCLR, Nanosensors, Switzerland). The cantilever of the tip (normal spring constant of 68 N/m) was made of silicon (Fig. 1b), and a silicon tip (radius of 10 nm) (TESPA, Bruker Company, Germany) was employed to measure the grooves after machining.

### Fabrication of nanochannel and microchannel molds

**Fig. 2:**
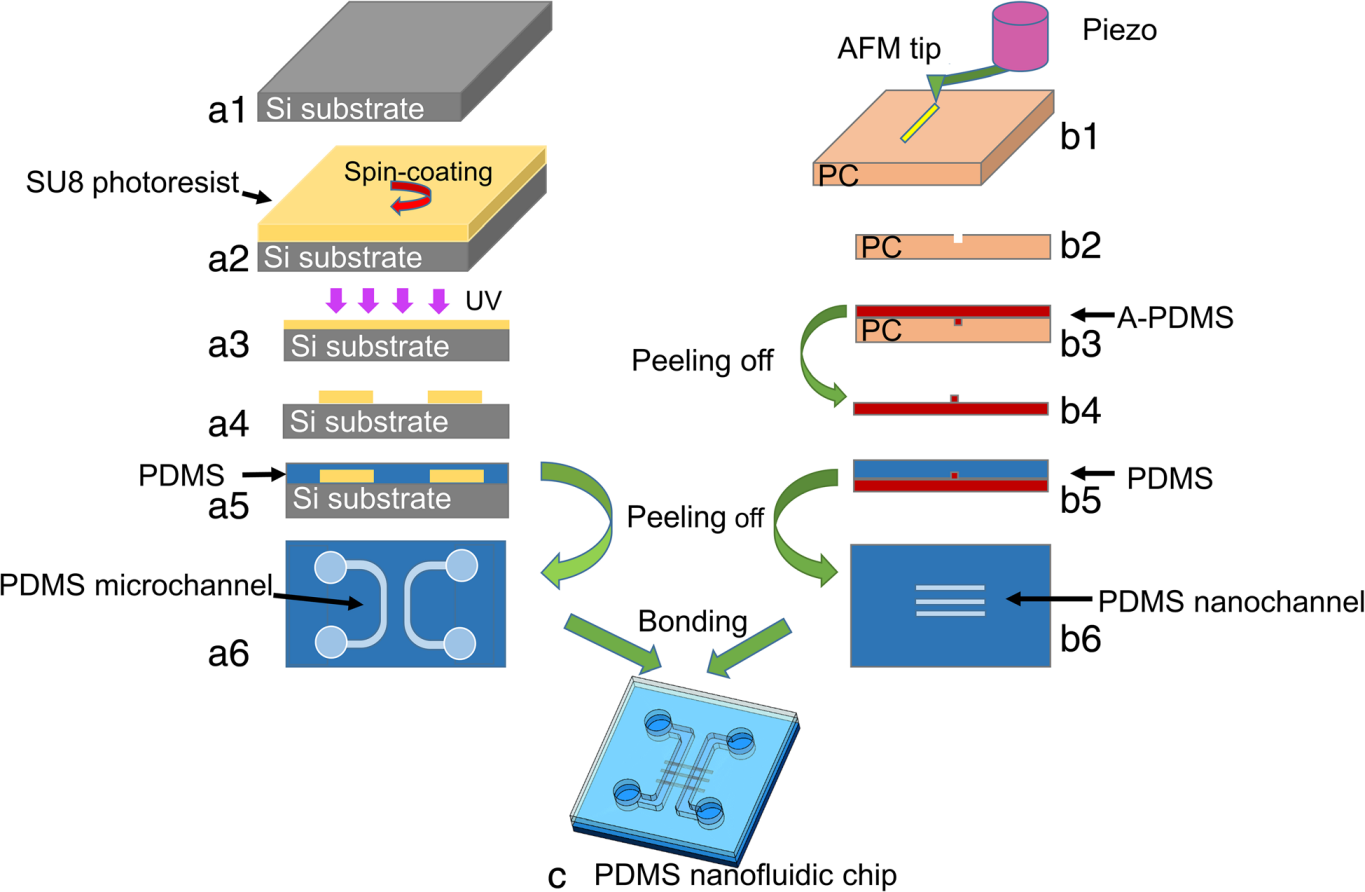
Flowchart of nanofluidic chip fabrication: (a1)–(a6) Working steps of microchannel fabrication on a PDMS chip; (a1) Silicon sheet used for lithography substrate; (a2) Spin-coating of SU8 photoresist on Si substrate; (a3) Exposure of the SU8 layer to UV light; (a4) Obtained convex microstructures; (a5) PDMS coating on microchannel mould; (a6) Final PDMS chip with microchannels; (b1)–(b2) Working steps of nanochannel fabrication on a PDMS chip; (b1) AFM tip scratches on PC sheet; (b2) Obtained nanochannel mould after scratching; (b3) A-PDMS coating on nanochannel mould; (b4) A-PDMS chip with convex nanostructures; (b5) Regular PDMS coating on A-PDMS mould; (b6) Final PDMS chip with nanochannels; (c) PDMS nanofluidic chip after bonding

The fabrication route of nanochannels on a PDMS chip is displayed in Fig. 2. The AFM system and the piezoelectric actuator were employed to fabricate nanochannel molds (with controllable sizes) on PC sheet. The PC sheet (molecular weight of 35,000) of 15 mm × 12 mm × 1 mm size was purchased from Goodfellow. The mean and the standard deviation of surface roughness (Ra) of the PC sheet were measured as 0.6 nm and 0.2 nm, respectively (these values were obtained by scanning a 50 μm × 50 μm area of the sample in AFM tapping mode). In order to generate a circular motion, the piezoelectric actuator was driven by sinusoid signals with 90° phase difference in x- and y-directions. The widths of the machined nanochannels depended on the amplitude of the generated circular motion. The range of the driving voltage inputted to the piezoelectric actuator was set from 30 V to 150 V with a spacing of 30 V, and in addition, two diving frequencies of 100 Hz and 1500 Hz were selected. During machining along the edge-forward direction, materials are expelled in pile-up formation and are often found to be uniformly distributed on both sides of a nanochannel [45], and it helps in avoiding any leakage of nanofluidic chips during bonding process; therefore, the edge-forward machining direction was selected in the present study. Nanochannels of 80-μm length were fabricated using the Nanoman module of the AFM system. Any machining process is affected by feed value; hence, in order to eliminate this influence, feed rate should be varied with driving frequency. In the present study, the feed value was set to 10 nm, and the feed rates for 100 Hz and 1500 Hz frequencies were calculated as 1 μm/s and 15 μm/s, respectively. The normal load of the tip depended on the output voltage generated from the position-sensitive photodetector (PSD); thus, different normal loads used in our study were achieved by setting a relative voltage (setpoint). According to our previous work [46], machining normal load was calculated by Eq. (1) and sensitivity was measured from the slope of the obtained force-distance curve [47].

1 $$ {F}_{\mathrm{N}}={V}_{\mathrm{setpoint}}\times sensitivity\times {K}_{\mathrm{N}} $$

Hence, the normal loads for nanomilling process were set as 17 μN and 25 μN. Further, for comparison, nanochannel molds on PC sheet were also fabricated without vibration, this method is called single scratching. The normal loads for the single scratching process were set as 25 μN, 33 μN, 42 μN, 50 μN, and 58 μN. The schematic diagram of nanochannel mold cross-section is displayed in Fig. 2(b2).

Microchannel molds were prepared by UV lithography process. The flowchart in Fig. 2(a1–a4) depicts the operation details of lithography process. The photoresist (SU-82015; MicroChem, USA) was spin-coated on Si substrate at 500 rps for 30 s and at 4000 rps for 120 s. A pair of “U”-shaped microchannels formed the microchannel chip (Fig. 2(a6)), which was bridged by nanochannels to form the final nanofluidic chip. The width of the microchannel was 30 μm and the diameter of the reservoir was 1 mm. Moreover, the distance between two “U”-shaped microchannels was 50 μm (Additional file 1: Figures S1 and S2).

### Transfer printing of microchannels and nanochannels

The convex microchannel mold (Fig. 2(a4)) and the concave nanochannel mold (Fig. 2(b2)) were transferred by PDMS (Sylgard 184, Dow Corining, USA) to prepare the final nanofluidic chip. Figure 2 (b3)–(b6) present the technological process of nanochannel mold transfer, which consisted of two steps: first transfer and second transfer. To investigate the effects of the weight ratio of monomer to curing agent on nanochannel size, three different PDMS weight ratios (A-PDMS) were employed during both first and second transfer processes. The PDMS weight ratios for the first transfer printing process were set as 9:1, 7:1, and 5:1, whereas the values for the second transfer were set as 10:1, 9:1, and 8:1. Figure 2(a5) and (a6) displays the transfer process of microchannel mold using one-step transfer approach. The PDMS weight ratio of 10:1 was used for the transfer of convex microchannel. During all of the transfer printing processes, two-component PDMS elastomer was first uniformly stirred and then poured into a case to prepare the mold. The case was then kept in a vacuum desiccator for 30 min and degassed for 2–3 times to remove all of the trapped air bubbles. The prepared mold was kept in a heating oven at 80 °C for 4 h, and finally, the PDMS replica was gently peeled off from the mold.

### Chip bonding

**Fig. 3:**
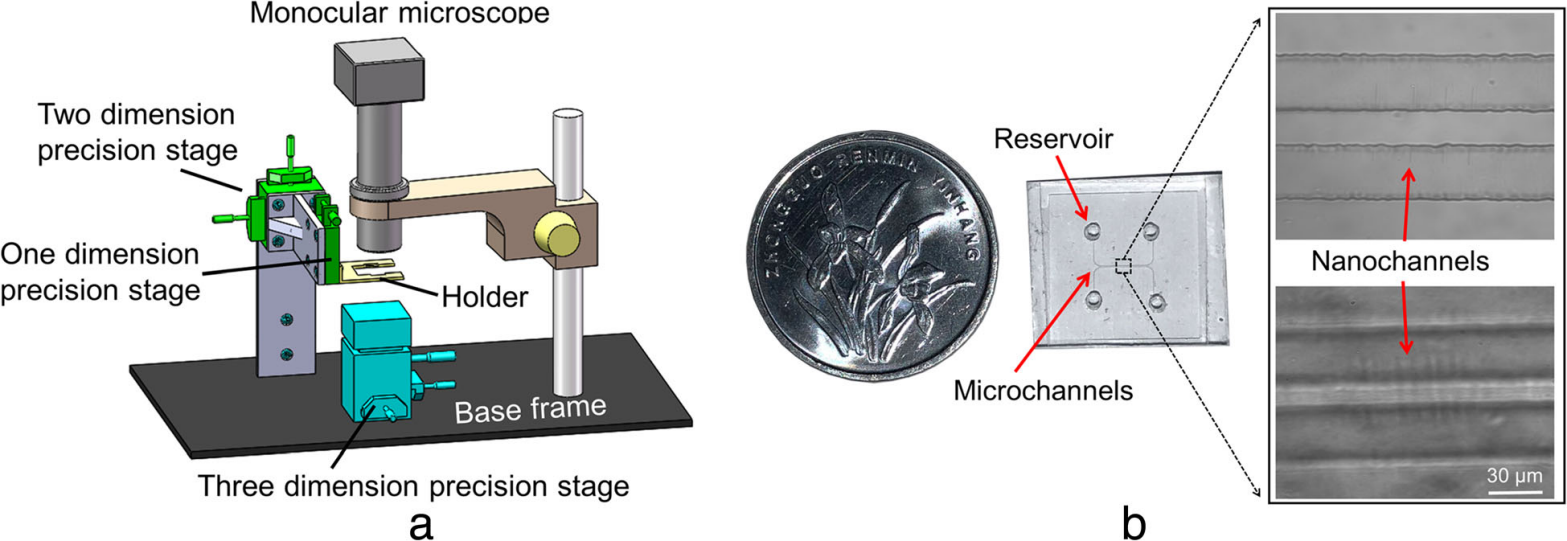
**a** Schematic diagram of homemade alignment system and **b** nanofluidic chip

The prepared nanofluidic chips were bonded by oxygen-plasma treatment (Zepto, Diener electronic, Germany) for a duration of 32 s under a chamber pressure of 1.5 mbar and a chamber power of 81 W (Fig. 2(c)). The surfaces of microchannels and nanochannels were cleaned by scotch tapes, and the four reservoirs on PDMS microchannel chips were punched before bonding. Deionized water was used to keep the chips clean after plasma treatment, and the chips were kept aligned together using a homemade alignment system that consisted of a holder, a monocular microscope, and a one-dimensional precision stage (TSDT-401S; SIGMAKOKI, Japan) (Fig. 3a). The details of the homemade alignment system can be found in the ESI. The chips were then bonded at a temperature of 95 °C for 20 min in order to obtain an enclosed micro/nanochannel chip (Fig. 3b).

## Results and discussion

### Rotary trajectory of piezoelectric actuator

The two-dimensional piezoelectric actuator is a critical component to conduct rotary motion in an AFM tip-based nanomilling system. Therefore, to characterize its motions under a range of driving voltages and frequencies, preliminary scratching tests were carried out. Under the contact model with a scan range of 0 nm, the AFM tip first approached the surface of PC sheet under a given normal load and was kept static. The rotation of the two-dimensional piezoelectric actuator was controlled by a pre-set frequency and voltage. After the completion of scratching process, the AFM tip was lifted up from the surface of PC sheet. Thus the motion amplitude of the piezoelectric actuator was obtained as a function of driving voltage and frequency. The driving voltages were set in the range of 30–150 V with a spacing of 30 V, whereas the driving frequencies were set as 100 Hz and 1500 Hz. The relationship between measured amplitudes and driving voltages at two driving frequencies is displayed in Additional file 1: Figure S3. It is evident that the values of machining amplitude increased with the increasing driving voltages, and the value of machining amplitude at 1500 Hz was greater than that of 100 Hz. It was found that the widths of the nanochannel fabricated by our proposed method ranged from 350 nm to 690 nm.

### Fabrication of nanochannel molds on PC sheet

**Fig. 4:**
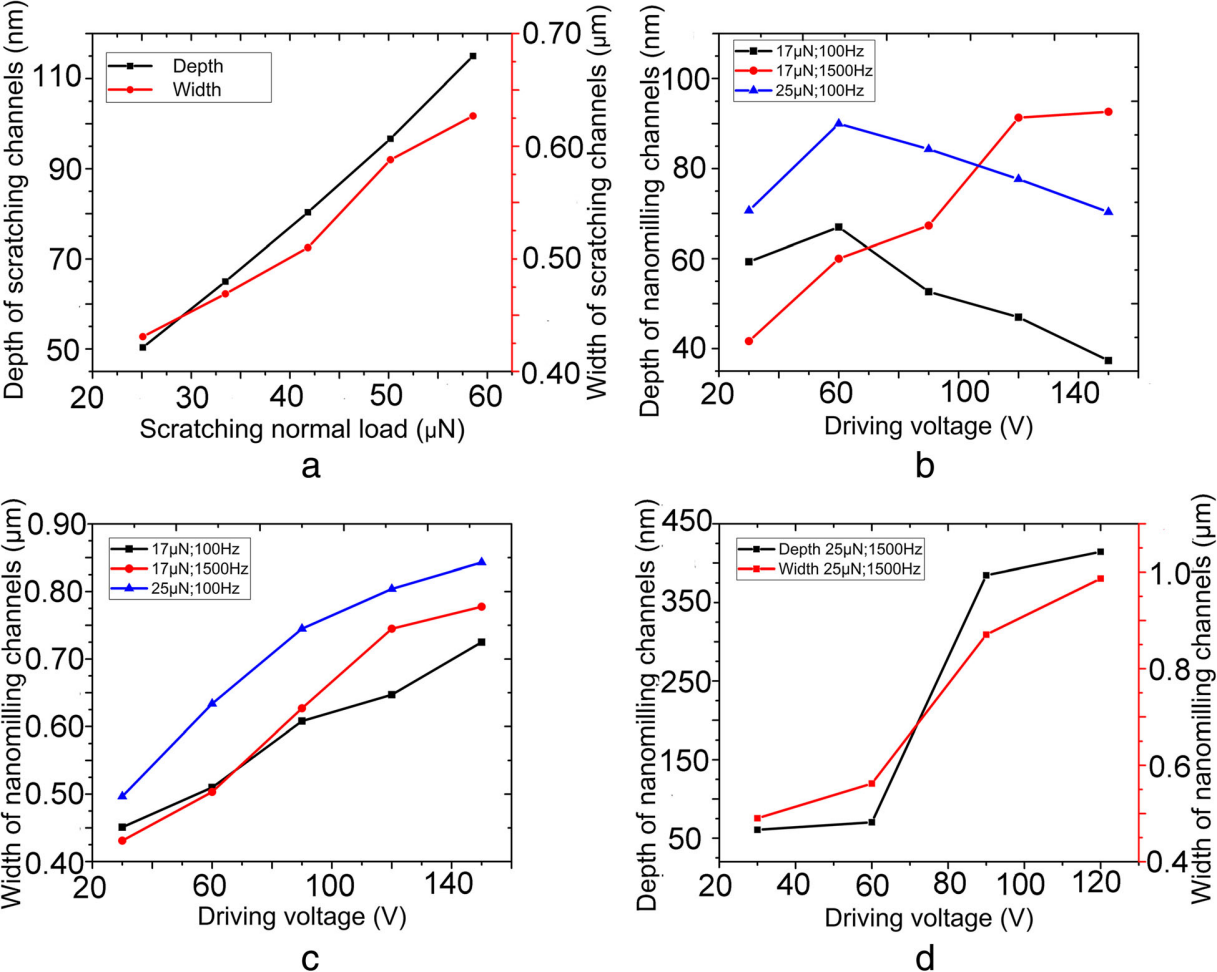
Relationship between machined nanochannel size and machining parameters: **a** single scratching with normal loads range from 25 μN to 58 μN, **b** depth, and **c** width of the machined channels when fabricated with normal loads of 17 μN, 25 μN and driving frequencies of 100 Hz, 150 Hz, **d** the depth and width of the machined channels when fabricated with normal load of 25 μN and driving frequency of 1500 Hz

**Fig. 5:**
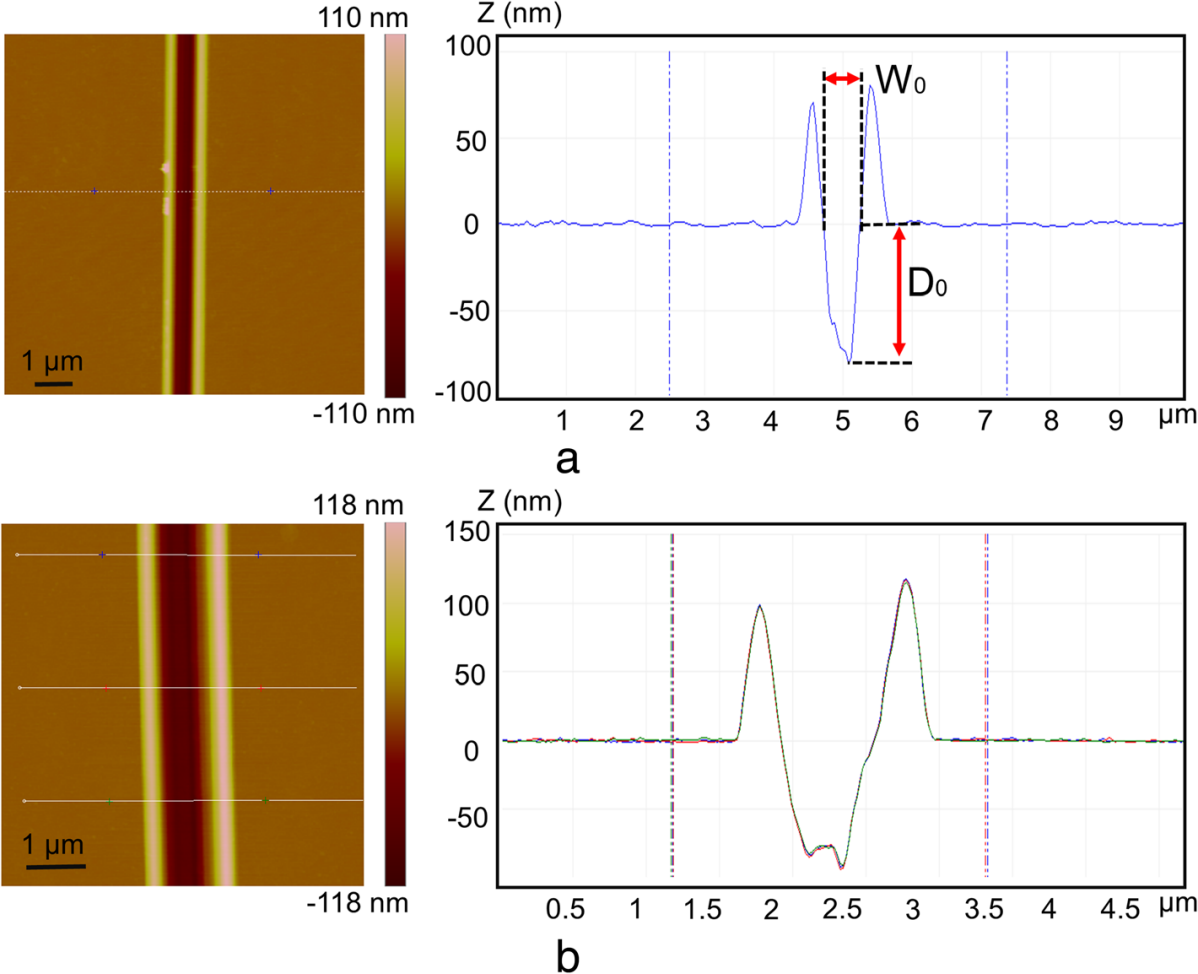
Typical AFM images of the machined nanochannel with different machining parameters: **a** Scratching under a normal load of 42 μN. **b** Nanomilling under a normal load of 25 μN, a frequency of 100 Hz, and a driving voltage of 60 V

The relationships between nanochannel size and machining parameters under single scratching and nanomilling are presented in Fig. 4a and b, respectively. The widths and the depths of the machined nanochannels are represented by *W*_0_ and *D*_0_, respectively (Fig. 5a).

It is observable from Fig. 4a that the widths and the depths of the fabricated nanochannels increased with the increasing normal loads. The typical AFM image of scratching under a normal load of 42 μN is exhibited in Fig. 5a. It is noticeable that materials were expelled from the nanochannel to form pile-ups, which were uniformly distributed on both sides of the nanochannel. Because the shape of AFM tip is symmetrical to the surface that was formed by edge “OA” during machining process (Fig. 1b). Thus, materials were expelled uniformly by the front edge of the tip during edge-forward scratching. Figure 4b, c, and d illustrate the relationship between machined nanochannel size and driving voltage. It is evident from Fig. 4b that the depth of the nanochannel increased at the beginning and then started to decrease under a frequency of 100 Hz with normal loads of 17 μN and 25 μN. The PC sheet used in our study was an amorphous polymer, which presents an elastic-viscoplastic behavior in combination of an exponential strain hardening at high-strain levels [48, 49]. The normal load during machining was calculated by Eq. (2), where $\overrightarrow{n}$ and $\overrightarrow{t}$ are the unit normal and the unit tangent to the flow line vector, respectively, *p* and *τ* signifies local normal pressure and shear stress, respectively, and $\overrightarrow{z}$ is the unit vertical [50].

2 $$ {F}_{\mathrm{N}}=p\cdot \int \overrightarrow{n}\cdot \overrightarrow{z} ds-\tau \cdot \int \overrightarrow{t}\cdot \overrightarrow{zds} $$

In the present study, the dimensional sizes of the fabricated nanochannels were found at nanoscale, thus the values of local normal pressure and shear stress were assumed constant. Further, Eq. (2) was converted into the simplified form of Eq. (3), where *S*_*n*_ and *S*_*h*_ respectively are the horizontal and the vertical projections of the interface between AFM tip and sample.

3 $$ {F}_{\mathrm{N}}=p\cdot {S}_n-\tau \cdot {S}_h $$

The relationship between *S*_*n*_ and *S*_*h*_ is expressed in Eq. (4), where *α* and *β* respectively are the included angles between tip surface and vertical and horizontal planes.

4 $$ {S}_{\mathrm{n}}=\frac{S_{\mathrm{h}}}{\cos \alpha}\cdot \cos \beta $$

The normal load was calculated by Eq. (5).

5 $$ {F}_{\mathrm{N}}=\left(p\cdot \frac{\cos \beta }{\cos \alpha }-\tau \right)\cdot {S}_h $$

It is evident from Eq. (1) that the values of normal load were constant during the entire machining process. According to Briscoe et al. [51], the value of mean strain rate was calculated by Eq. (6), where *V* and *w* signify tip speed and uncut chip thickness, respectively. The maximum value of uncut chip thickness was found as ~ 10 nm.

6 $$ {}_{\varepsilon}^{\bullet }=\frac{\mathrm{d}\varepsilon }{\mathrm{d}t}\approx \frac{V}{w} $$

Moreover, the values of tip speed were obtained from Eq. (7), where *f* is input signal frequency.

7 $$ V=\pi \cdot {W}_o\cdot f $$

The values of mean strain rate at 100 Hz were found in the range of 1.42 × 10^4^ s^-1^~ 2.27 × 10^4^ s^-1^. The values of local normal pressure (*p*) started to go up with the increasing of strain rates when the strain rates ranged from 1.42 × 10^4^ s^-1^ to 2.27 × 10^4^ s^-1^ [52]. The value of *τ* was much smaller than that of *p*, it signifies that normal load mainly depended on *p*. Therefore, in order to keep the values of normal load (F_*N*_) constant during the entire machining process, the values of machining depth should be smaller at higher driving voltages. However, the final dimensional size of the fabricated nanochannel was affected by the recovery of the sample material. The recovery of the sample decreased with the increasing machining speeds in the range of 142~227 μm/s [53]: thus, it indicates that a higher elastic recovery occurred at 30 V. Consequently, the depth of the fabricated nanochannel at 30 V (~142 μm/s) was shallower than that of 60 V (~161 μm/s). Additional file 1: Figure S4(a) and Fig. 5b are the typical AFM images of the nanochannel machined at 100 Hz under normal loads of 17 μN and 25 μN, respectively. It is obvious that the pile-up at the right side of the nanochannel is larger than the left. The rotary motion of the sample during nanomilling process is anticlockwise, and the cutting angle of the main cutting edge is changing with the rotation. The uncut chip thickness is too small to form chips at the beginning and end of a cycle of nanomilling process. The uncut chip thickness at the middle of a cycle of nanomilling process is relatively large; however, the small attack angle contributes to the formation of the pile-ups. Thus, more materials are pushed to the right side of the channel, and the pile-ups are thus asymmetric*.* The details for the formation of asymmetric pile-ups can be found in our previous study [54].

**Fig. 6:**
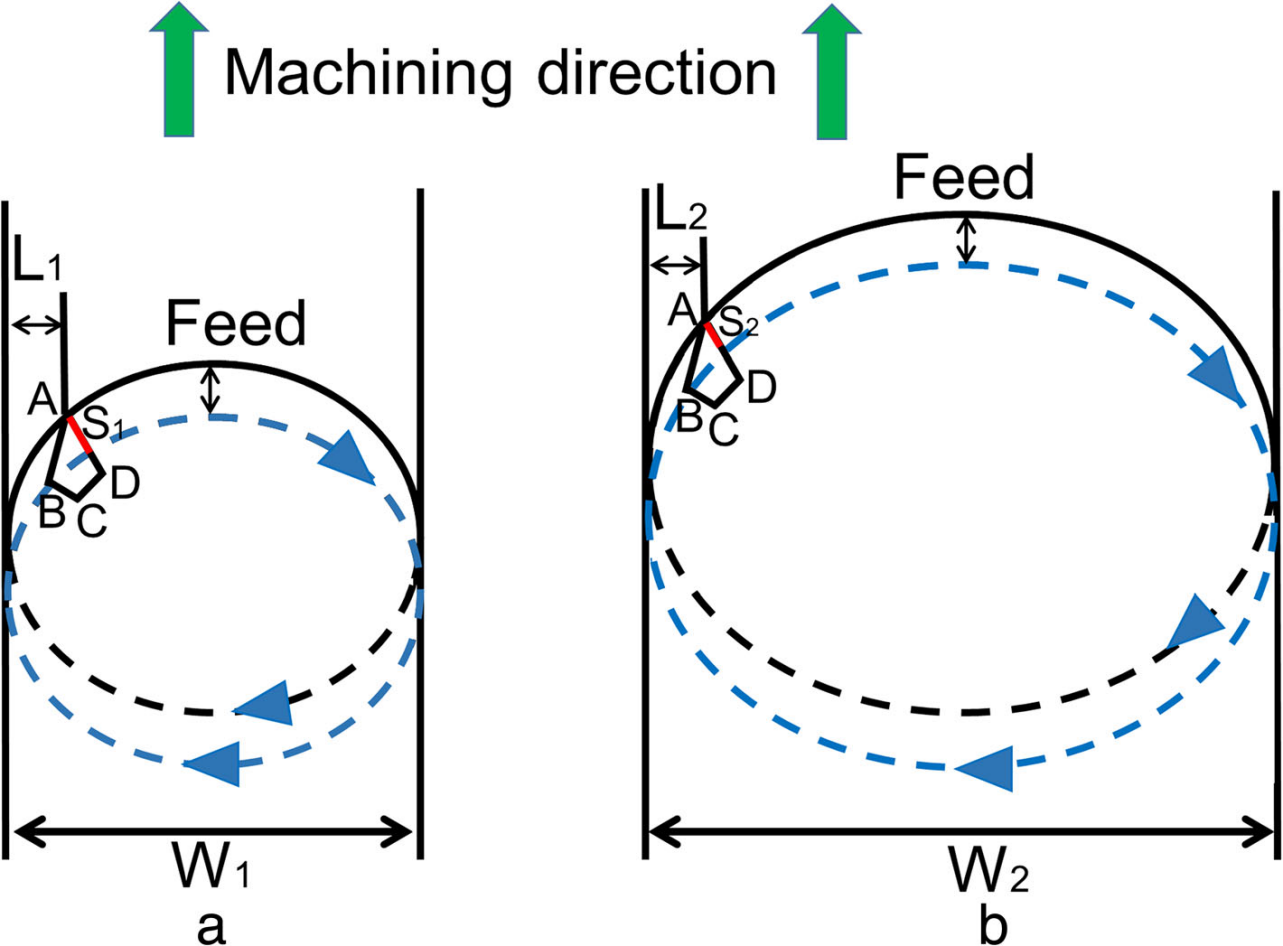
Schematic representation of AFM tip trajectory during nanomilling process: the machined width of nanochannel (**a**) is smaller than nanochannel (**b**), and the dashed ellipses, the black solid ellipses, and the blue arrows represent the finished machining process, the on-going machining process, and the motion direction of AFM tip, respectively

It is observable from Fig. 4b and d that the depth of the nanochannel started to increase with the increasing driving voltages at 1500 Hz under normal loads of 17 μN and 25 μN. Figure 4d depicts that nanochannel depth increased sharply from 60 V (~ 2.64 mm/s) to 90 V (~ 4.10 mm/s) under a normal load of 25 μN. According to Geng et al. [55], material removal state is significantly affected by cutting speed. Materials were expelled from the nanochannel in pile-up form during machining at a speed of 2.64 mm/s, whereas material removal state was changed from pile-up to chip at 4.10 mm/s (Additional file 1: Figure S4(b)). Therefore, the increase in machining depth at 90 V (~4.10 mm/s) can be attributed to the change in material removal state. The width of the fabricated nanochannel started to increase with the increasing driving voltages. Figure 6 displays the schematic diagram of AFM tip trajectory during nanomilling, the dashed ellipses, the black solid ellipses, and the blue arrows represent the finished machining process, the on-going machining process, and the motion direction of AFM tip, respectively. The width (*W*_2_) of the machined channel in Fig. 6(b) was larger than that (*W*_1_) in Fig. 6(a). AS_1_ and AS_2_ (red solid lines) represent the contact length between the cross-section of AFM tip and sample material. The value of AS_1_ was found to be greater than that of AS_2_ when the machining width “*L*_1_” was equal to “*L*_2._”

The value of S_*h*_ in Eq. (5) was obtained by Eq. (8), where D and AS respectively are the depth of the machined channel and the contact length between the cross-section of AFM tip and sample material.

8 $$ {S}_h=\frac{1}{2}\cdot \left|D\left|\cdot \right|\; AS\right| $$

Hence, Eq. (5) was further rewritten in the form of Eq. (9).

9 $$ {F}_{\mathrm{N}}=\frac{1}{2}\cdot \left(p\cdot \frac{\cos \beta }{\cos \alpha }-\tau \right)\cdot \left|D\left|\cdot \right| AS\right| $$

The values of *α* and *β* were kept constant during the entire machining process. The values of strain rate at 1500 Hz were found in the range of 2.03 × 10^5^~3.66 × 10^5^ s^-1^; hence, it can be assumed that local normal pressure (*p*) reached its limit value at 1500 Hz. Furthermore, machining speed manifested no effect on the recovery of the sample during machining at 30– 150 V (~ 2.03–3.66 mm/s) [53]; thus, the final dimensional sizes of the nanochannel were only determined by machining dimensions. The values of AS_2_ (Fig. 6(b)) were found to be smaller than those of AS_1_ (Fig. 6(a)) for larger machined widths, and according to Eq. (9), the value of D was larger for a smaller value of AS. Therefore, the values of machining depth increased with the increasing driving voltages. A typical AFM image of the nanochannel fabricated under a normal load of 25 μN, a driving voltage of 120 V, and a frequency of 1500 Hz is presented in Additional file 1: Figure S4(b). It is noticeable that materials were removed in both chip and pile-up formation, and the expelled materials accumulated only on one side of the nanochannel. Moreover, the expelled materials accumulated in chip formation at the bottom of the nanochannel during machining at 150 V under a normal load of 25 μN. Therefore, the size data of the fabricated nanochannel during machining at a voltage of 150 V and a frequency of 1500 Hz (under a normal load of 25 μN) was empty in Fig. 4d.

It is evident from Fig. 4c that nanochannel width started to increase with the increasing driving voltages. Moreover, when the values of normal load and driving voltage were kept constant, the width of the nanochannel fabricated at a frequency of 1500 Hz was wider than that of 100 Hz. Moreover, machining depth of the nanochannel fabricated at 1500 Hz was deeper than that of 100 Hz, and the cross-sectional size of the tip was found to be larger during the machining of a deeper nanochannel. Therefore, the nanochannels were fabricated wider when machining deeper.

### First transfer of nanochannel molds

Nanochannels machined by single scratching method under normal loads of 25 μN, 33 μN, 41 μN, 50 μN, and 58 μN were applied to the first transfer process. Moreover, nanochannel molds fabricated by nanomilling at a frequency of 100 Hz in the driving voltage range of 30–150 V (with a spacing of 30 V) were also used in the transfer process. Nanochannels (80 nm depth and 510 nm width) machined by single scratching method were termed as “nanochannel I”, whereas nanochannels (50 nm depth and 610 nm width, 90 nm depth and 630 nm width) fabricated by nanomilling were called as “nanochannel II” and “nanochannel III,” respectively. Three different PDMS weight ratios (5:1, 7:1, and 9:1) were used in the first transfer process.

**Fig. 7:**
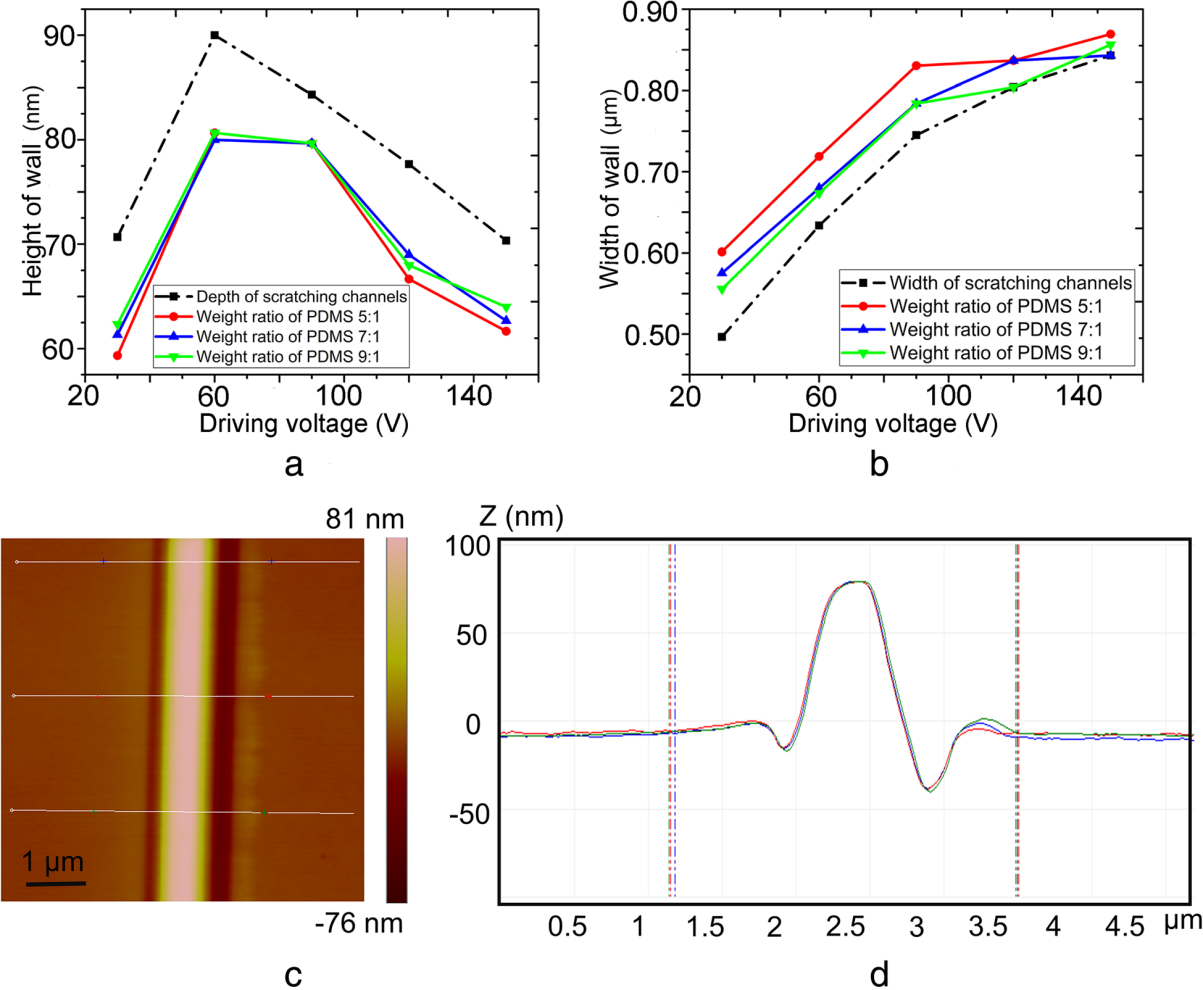
Relationship between **a** wall height, **b** wall width, and transfer parameters (various weight ratio of PDMS) during first transfer process, where the channel molds were fabricated with a normal load of 25 μN and a frequency of 100 Hz, and **c** typical AFM image and **d** corresponding cross-section of the wall obtained from nanochannel III at a weight ratio of 5:1

Figure 7a and b reveal the effects of different PDMS weight ratios on wall size under a normal load of 25 μN and a frequency of 100 Hz, and the black dash line represents the original nanochannel size before transfer. The typical AFM image and corresponding cross-section of the wall obtained from nanochannel III at a weight ratio of 5:1 during first transfer are displayed in Fig. 7c and d, and this wall was termed as “wall III.” The effects of different PDMS weight ratios on the wall size under single scratching process with a normal load of 17 μN and a frequency of 100 Hz were shown in ESI (see Additional file 1: Figures. S5, S6, S7, and S8 of ESI for details). The walls obtained from “nanochannel I” and “nanochannel II” were termed as “wall I” and “wall II,” respectively. It is evident that the heights of all walls at different PDMS weight ratios were approximately the same. The widths of the walls were larger than the original nanochannel width, and the width at the weight ratio of 5:1 was found to be the largest. Due to the thermal expansion of PC sheet, a small deviation was noticed between final wall size and original nanochannel size. It was also observed that the elasticity of PDMS increased as the PDMS weight ratio decreased from 5:1 to 7:1 [41, 42]. Hence, the wall obtained at the weight ratio of 5:1 was stiffer and its elastic recovery was smaller; thus, the width of the wall obtained at the weight ratio of 5:1 was the largest.

Second transfer of nanochannel molds

**Fig. 8:**
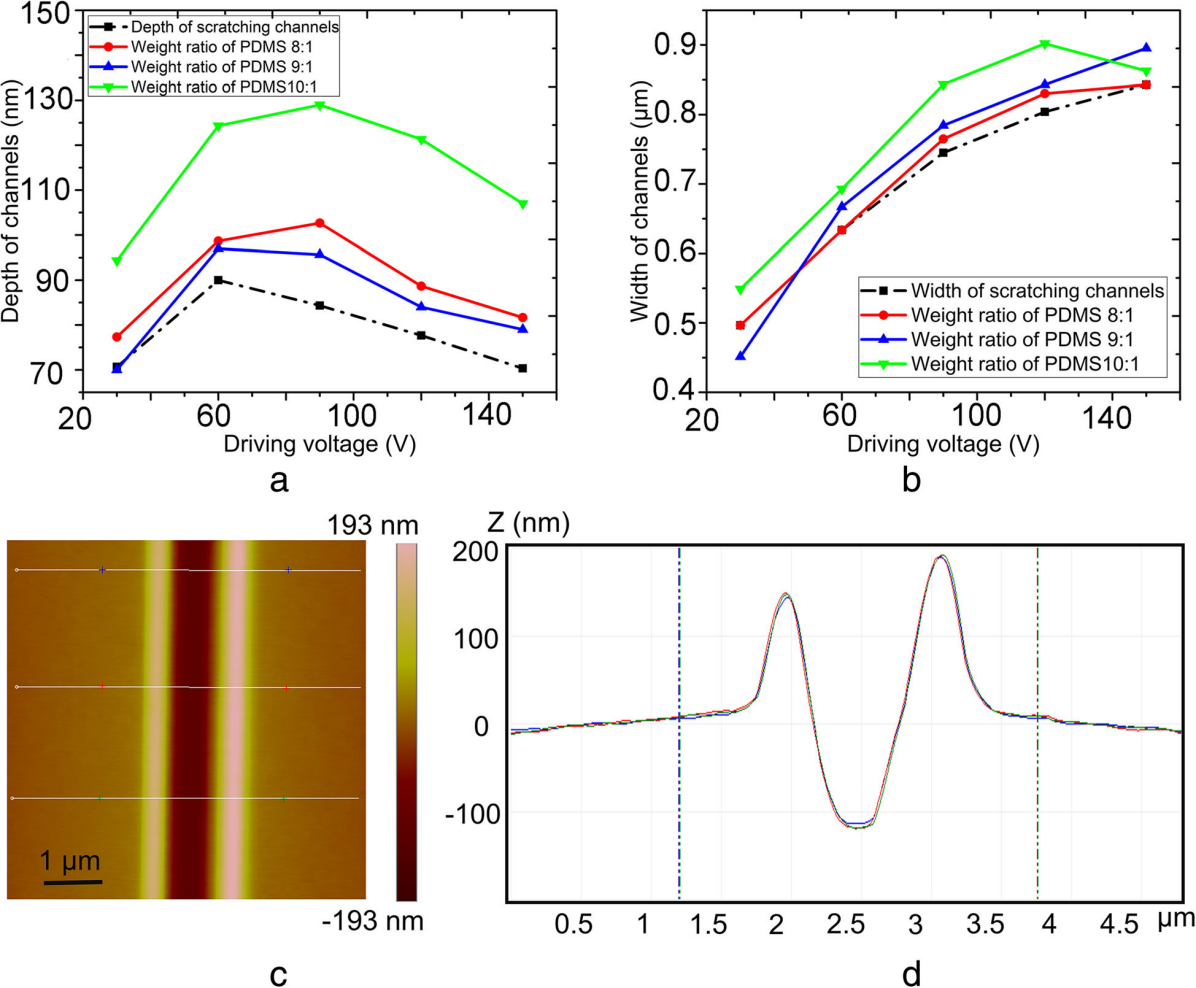
Relationship between **a** nanochannel height, **b** nanochannel width, and transfer parameters (various weight ratio of PDMS) during second transfer, where the channel molds were fabricated with a normal load of 25 μN and a frequency of 100 Hz, and **c** typical AFM image and **d** corresponding cross-section of the nanochannel obtained from wall III at a weight ratio of 10:1 during second transfer

The final PDMS slabs with nanochannels were obtained during second transfer process based on the wall obtained at a weight ratio of 5:1 in the first transfer process. Three different PDMS weight ratios (10:1, 9:1, and 8:1) were used during second transfer process. Figure 8a and b present the relationship between nanochannel size obtained under a normal load of 25 μN and a frequency of 100 Hz and transfer parameters during second transfer. It is clear from Fig. 8a that the depths of the nanochannels were larger than the original machining size, moreover, the depth at 10:1 was found to be larger than other two ratios. Further, the widths of the wall were also larger than the original size, and the width at 10:1 was found to be the largest (Fig. 8b). Figure 8c and d present a typical AFM image and corresponding cross-section of the nanochannel (120 nm depth and 690 nm width) obtained from wall III at a weight ratio of 10:1 during second transfer, and it was termed as “nanochannel C.” The relationship between the nanochannel sizes obtained under single scratching process with a normal load of 25 μN and a frequency of 100 Hz and the transfer parameters during the second transfer process were shown in ESI (see Additional file 1: Figures. S9, S10, S11 and S12 of ESI for details), the nanochannels obtained from “wall I” and “wall II” were termed as “nanochannel A” and “nanochannel B”, respectively.

The depths of nanochannels obtained from walls II and III were larger than the original machining size, whereas the depth obtained from wall I was smaller than the initial machining size. Furthermore, the changes in width were identical to the changes in depth. The aspect ratio of wall I was larger than those of walls II and III, thus each wall manifested different thermal expansion values. Hence, the changing trends of width and depth during second transfer were different though at the same PDMS weight ratio. The values of the depth and width of walls II and III at 9:1 and 8:1 were found to be closer to the original machining size compared with 10:1. Because the elastic recoveries of PDMS at 9:1and 8:1 are closer to 5:1 than 10:1, which indicates an almost similar recovery trend for PDMS at 9:1, 8:1, and 5:1.

### Application of nanochannel devices in electric current measurement

**Fig. 9:**
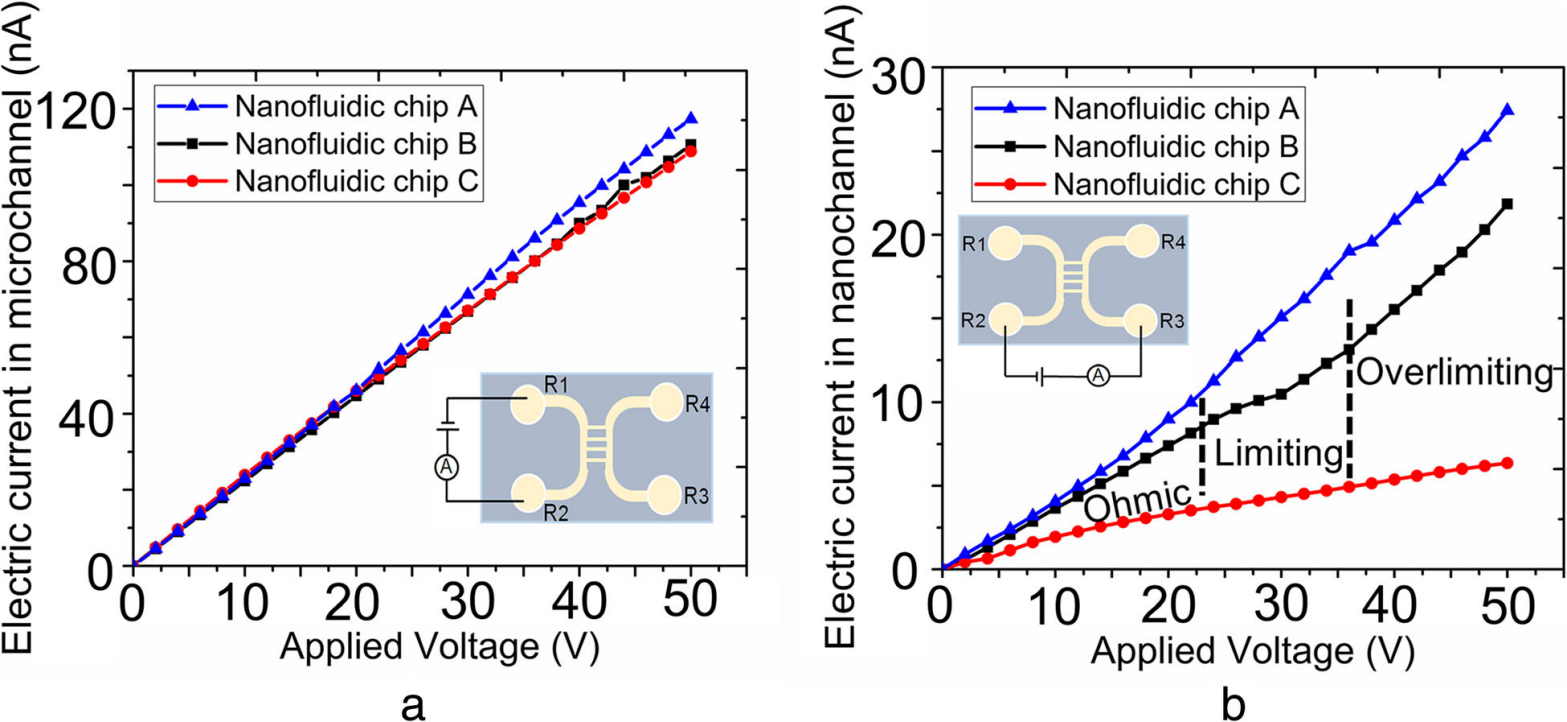
Electric current measurement results based on the fabricated nanochannel devices, the cross-section size (depth × width) of nanochannels for nanofluidic chip A, B, and C are 60 × 500 nm, 80 × 680 nm and 120 × 690 nm, respectively. **a** Current in microchannels. **b** Current in nanochannels. The insets display the schematic sketches of the measurements

Nanochannel devices are often used in the fields of single nanoparticle manipulation, electrokinetic transport phenomena, DNA analysis, and enzymatic reaction detection. The main working principle of nanofluidic chips depends on the variation in electric current; therefore, it is important to measure the electrical conductivities of nanochannel devices. The electrical conductance in a nanochannel can be estimated by Eq. (10) [56].

10 $$ G={10}^3N\;{}_Ae\frac{wh}{l}\sum {\mu}_i{c}_i+2{\mu}_e\frac{w}{l}{\delta}_n $$

where *μ*_*i*_ is the mobility of ion *i*, *c*_*i*_ is the concentration of ion *i*, *δ*_*n*_ is the effective surface charge inside the nanochannel, and N_*A*_ and *e* signify Avogadro constant and electron charge, besides, *w*, *h* and *l* are the nanochannel width, height and length, respectively. It is obvious that the electrical conductance of a nanochannel is affected by the nanochannel feature dimensions and the solution concentration. The electric double layer (EDL) plays an important role in the nanochannel when the ratio of DEL thickness to the nanochannel height increases. The diffuse layer thickness of EDL is 3~5 times of the Debye length (*λ*_*D*_), which can be expressed by Eq. (11) [57].

11 $$ {\lambda}_D=\sqrt{\frac{\varepsilon_0{\varepsilon}_r{k}_bT}{2{n}_{i\infty }{(ze)}^2}} $$

where *n*_*i*∞_ denotes ion density in the solution, *ε*_*o*_ is the permittivity of vacuum, *ε*_*r*_ is the dielectric constant of electrolyte solution, *z* is the valency of buffer solution (*z* = *z*^+^ − *z*^−^ = 1 for KCl), and k_*b*_ and *T* are the Boltzmann constant and temperature, respectively. In the present study, three different nanofluidic chips were obtained after the completion of transfer process. Nanofluidic chips consisted of nanochannels A, B, and C were termed as nanofluidic chips A, B, and C, respectively. Each nanofluidic chip contained four nanochannels. The widths and the depths of nanofluidic chips A, B, and C were measured as 60 nm and 500 nm, 80 nm and 680 nm, and 120 nm and 690 nm, respectively. The effective length of nanochannels in all chips was calculated as 50 μm. As shown in Fig. 8, pile-ups distribute on the sides of the nanochannels A, B, and C. The pile-ups may fill into the nanochannels and lead to a failure of the preparation for the nanofluidic chips. Thus, in order to verify the reliability of the fabricated nanochannel devices, electrical conductivity measurement test was conducted. KCl with 1 mM concentration was as the electrolyte solution in our study, and the values of electrical current were measured by an electrometer (Model 6430, Keithley, USA). The schematic sketches of the measurements for electric current in microchannel and nanochannel are presented as the inset figures in Fig. 9a and b, respectively. The experiments were carried out under DC power (applied by an Ag electrode) with an increment of 2 V for 3-s duration. Figure 9a presents the measured *I*-*V* curves of microchannels in three different nanofluidic chips, and a linear relationship between current and voltage was observed. Moreover, as the effect of EDL in microchannels was negligible and the dimensional sizes of microchannels in different nanofluidic devices were identical, the values of current in different chips were nearly the same. It is evident from Fig. 9b that the values of current in different nanofluidic devices were distinct due to different nanochannel sizes. For KCl solution of 1 mM concentration, the value of *λ*_*D*_ was about 10 nm, thus the diffuse layer thickness of EDL was found as 30~50 nm [57]. Consequently, EDL got overlapped along the depth (60 nm) of nanofluidic chip A; however, no overlapping was observed in nanofluidic chip C (depth of 120 nm). However, it was difficult to determine whether EDL got overlapped or not in nanofluidic chip B (depth of 80 nm). It assumes that the effective surface charges (*δ*_*n*_) in all nanochannels are identical as the charge density of a surface is material property [58, 59]. The concentration of the ions in a nanochannel depends on the EDL field, the stronger the EDL field, the higher the ion concentration in the nanochanel [44]. In the present study, the EDL field in nanofluidic chip A is the strongest as the highest ratio of the DEL thickness to the nanochannel height, which signifies that the ion concentration in the nanochannel of nanofluidic chip A is the highest. According to Eq. (10), the nanochannel of nanofluidic chip A is more conductive due to the higher ion concentration. Hence, the value of electrical current in nanofluidic chip A was the largest, whereas nanofluidic chip C yielded the smallest value. In addition, at larger width sizes, EDLs did not overlap along the width directions of nanochannels. In nanofluidic chip B, when the value of applied electric field was lower than 25 V, a linear relationship was noticed between current and applied voltage; however, a limiting region appeared as the value of applied voltage increased and finally, became liner again as the electrical field increased further, this phenomenon belongs to ohmic-limiting-overlimiting current characteristic [60, 61]. The results of electrical current measurement revealed that the nanofluidic devices fabricated by the proposed method were effective, the pile-ups of the nanochannels A, B, and C had almost no influence on the performance of the nanofluidic devices.

## Conclusions

In the present research, nanochannels with controllable sizes (sub-100-nm depth) were fabricated by AFM tip-based nanomilling, and for the first time, the machined nanochannels were applied to prepare nanofluidic devices. The multichannel nanofluidic devices were prepared in four steps: (1) fabrication of nanochannels by AFM tip and piezoelectric actuator, (2) fabrication of microchannels by lithography, (3) transfer of micro- and nanochannels, and (iv) bonding. Further, nanochannel sizes were controlled by changing the driving voltages and frequencies inputted to the actuator. The heights of the wall obtained during first transfer were smaller than the original machining size, whereas the widths were larger than the original machining size. The experiment results revealed that during second transfer process, nanochannel sizes affected PDMS weight ratios. Finally, micro-nanofluidic chips with three different nanochannel sizes were obtained by bonding a PDMS nanochannel chip on a PDMS microchannel chip. Moreover, the electrical current measurement experiment was conducted on the fabricated nanofluidic chips, and it was found that the values of current were affected by nanochannel sizes. Therefore, PDMS nanofluidic devices with multiple nanochannels of sub-100-nm depth can be efficiently and economically fabricated by the proposed method.

Compared with other fabrication approach, the proposed method for fabrication of the nanofluidic devices in the study is easy to use and low cost; besides, the nanochannels with controllable dimension size can be obtained easily. However, the commercial AFM system cannot equip with a large-scale high-precision stage due to the spatial limitation; thus, the maximum fabrication length of the nanochannel is confined as 80 μm. In addition, the tip wear cannot be neglected after long-term fabrication due to the high machining speed, which should be investigated in future work.

## Additional file

Additional file 1: **Figure S1**. Schematic diagram of homemade alignment system. **Figure S2**. Schematic illustrations of alignment procedures during bonding process. **Figure S3**. Relationship between scratching circle diameter and driving voltage. **Figure S4**. Typical AFM images of the machined nanochannel with different machining parameters. **Figure S5**. Relationship between wall size and transfer parameters (various weight ratio of PDMS) during first transfer process, where the channel molds were fabricated with single scratching approach: (a) Wall height, (b) Wall width. **Figure S6**. Typical AFM image (left) and corresponding cross-section (right) of the wall obtained from nanochannel I at a PDMS weight ratio of 5:1 during first transfer. **Figure S7**. Relationship between wall size and transfer parameters (various weight ratio of PDMS) during first transfer process, where the channel molds were fabricated with a normal load of 17 μN and a frequency of 100 Hz: (a) Wall height, (b) Wall width. **Figure S8**. Typical AFM image (left) and corresponding cross-section (right) of the wall obtained from nanochannel II at a weight ratio of 5:1 during first transfer. **Figure S9**. Relationship between nanochannel size and transfer parameters (various weight ratio of PDMS) during second transfer, where the channel molds were fabricated with single scratching approach: (a) Nanochannel depth, (b) Nanochannel width. **Figure S10**. Typical AFM image (left) and corresponding cross-section (right) of the nanochannel obtained from wall I at a weight ratio of 10:1 during second transfer. **Figure S11**. Relationship between nanochannel size and transfer parameters (various weight ratio of PDMS) during second transfer, where the channel molds were fabricated with a normal load of 17 μN and a frequency of 100 Hz: (a) Nanochannel depth, (b) Nanochannel width. **Figure S12**. Typical AFM image (left) and corresponding cross-section (right) of the nanochannel obtained from wall II at a weight ratio of 10:1 during second transfer. (ZIP 16617 kb)

## Abbreviations

AFM: Atomic force microscope
DC: Direct current
EDL: Electric double layer
KCL: Potassium chloride
PC: Polycarbonate
PDMS: Polydimethylsiloxane
PSD: Position-sensitive photodetector
