# Supplementary material for: Fabrication of polydimethylsiloxane nanofluidic chips under AFM tip-based nanomilling process
Source: Nanoscale Res Lett. 2019 Apr 17;14:136. doi: 10.1186/s11671-019-2962-6 (PMC6470239; doi:10.1186/s11671-019-2962-6)
Supplement: Supplementary file 1 — Figure S1. Schematic diagram of homemade alignment system. Figure S2. Schematic illustrations of alignment procedures during bonding process. Figure S3. Relationship between scratching circle diameter and driving voltage. Figure S4. Typical AFM images of the machined nanochannel with different machining parameters. Figure S5. Relationship between wall size and transfer parameters (various weight ratio of PDMS) during first transfer process, where the channel molds were fabricated with single scratching approach: (a) Wall height, (b) Wall width. Figure S6. Typical AFM image (left) and corresponding cross-section (right) of the wall obtained from nanochannel I at a PDMS weight ratio of 5:1 during first transfer. Figure S7. Relationship between wall size and transfer parameters (various weight ratio of PDMS) during first transfer process, where the channel molds were fabricated with a normal load of 17 μN and a frequency of 100 Hz: (a) Wall height, (b) Wall width. Figure S8. Typical AFM image (left) and corresponding cross-section (right) of the wall obtained from nanochannel II at a weight ratio of 5:1 during first transfer. Figure S9. Relationship between nanochannel size and transfer parameters (various weight ratio of PDMS) during second transfer, where the channel molds were fabricated with single scratching approach: (a) Nanochannel depth, (b) Nanochannel width. Figure S10. Typical AFM image (left) and corresponding cross-section (right) of the nanochannel obtained from wall I at a weight ratio of 10:1 during second transfer. Figure S11. Relationship between nanochannel size and transfer parameters (various weight ratio of PDMS) during second transfer, where the channel molds were fabricated with a normal load of 17 μN and a frequency of 100 Hz: (a) Nanochannel depth, (b) Nanochannel width. Figure S12. Typical AFM image (left) and corresponding cross-section (right) of the nanochannel obtained from wall II at a weight ratio of 10:1 during second transfer. (ZIP 16 [file 11671_2019_2962_MOESM1_ESM.zip › Supplementary material.docx]

**Supplementary material**

**Fabrication of polydimethylsiloxane nanofluidic chips under AFM tip-based nanomilling process**

Jiqiang Wang^1,2^, Yongda Yan^1,2^, Yanquan Geng^1,2,3*^, Yang Gan^3^, Zhuo Fang^2^

*^1^ Key Laboratory of Micro-systems and Micro-structures Manufacturing of Ministry of Education, Harbin Institute of Technology, Harbin, Heilongjiang 150001, P.R. China*

*^2^ Center for Precision Engineering, Harbin Institute of Technology, Harbin, Heilongjiang 150001, P.R. China*

*^3^ School of Chemistry and Chemical Engineering, Harbin Institute of Technology, Harbin 150001, China*

** Corresponding author. Tel.: +86-0451-86412924. Fax: +86-0451-86415244*

*E-mail address:* [*gengyanquan@hit.edu.cn*](mailto:gengyanquan@hit.edu.cn) *(Y. Q. Geng)*

**Chip bonding**

Fig. S1 presents the schematic diagram of homemade alignment system, which consists of a holder, a monocular microscope and a one-dimensional precision stage (TSDT-401S, SIGMAKOKI, Japan). The one-dimensional precision stage and the two-dimensional precision stage are connected rigidly. Thus, the holder, which the microchannel chip is put on, can move freely in three directions. As shown in the local enlarging graph of Fig. S1, the PDMS blocks are fixed on the holder to avoid the microchannel chip sliding when conducting bounding process. The nanochannel chip is put on a glass slide and can move with the three-dimensional stage in three directions freely.


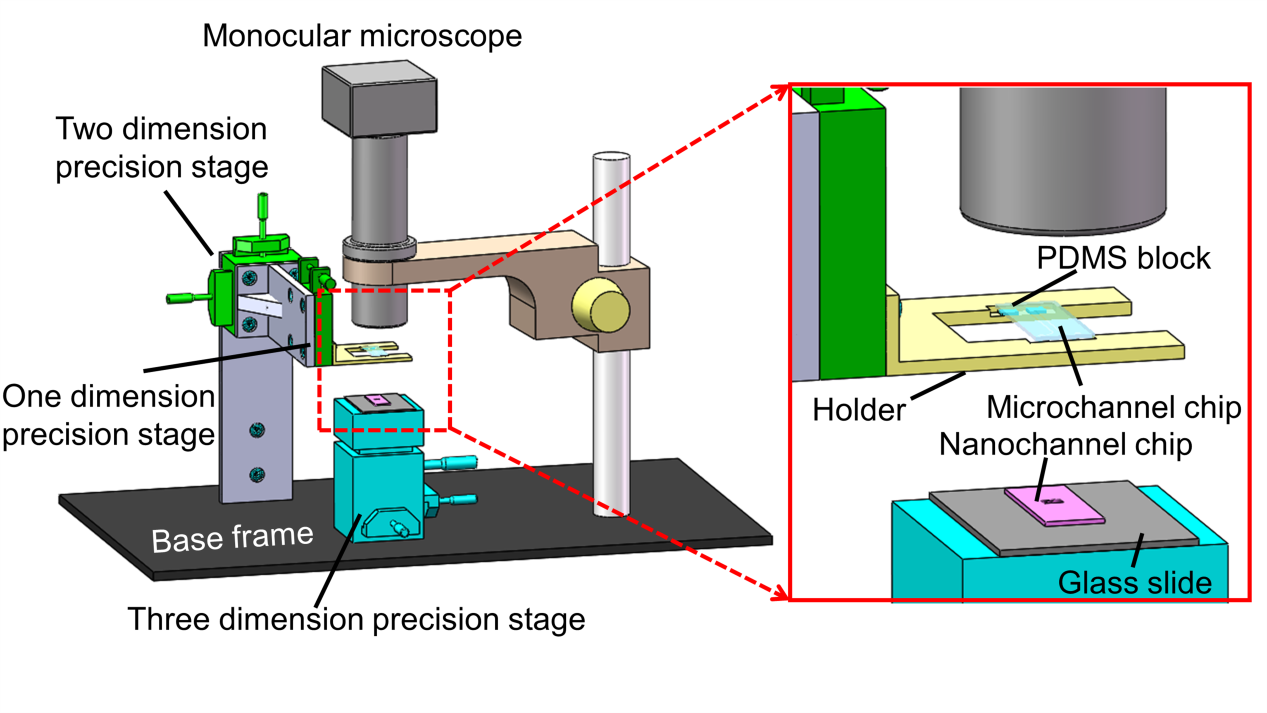


Fig. S1. Schematic diagram of homemade alignment system.

Fig. S2 exhibits the working principle of the homemade alignment system, where C is a nanochannel chip, and E is a microchannel chip. The bonding operation details can be processed in three steps. First, a nanochannel chip is placed onto the three-dimensional precision stage with nanochannels upwards, and the nanochannels are localized in the middle of the monocular microscope view under magnification of 16×. Second, put the microchannel chip on the proper position of the holder (B) with microchannel downwards, and the microchannels are located in the middle of the monocular microscope view under the same magnification. Finally, the microchannel chip is let down by the holder manually form the top until the microchannel chip is lifted by the nanochannel chip. Due to the properties of the plasma treated surface, the microchannel chip and nanochannel chip are bonded together automatically when the lower surface of microchannel chip touched the nanochannel chip upper surface. The glass slide (D), which the nanochannel chip is put on, works as a temporary substrate to move the bonded chip easily after bonding process.


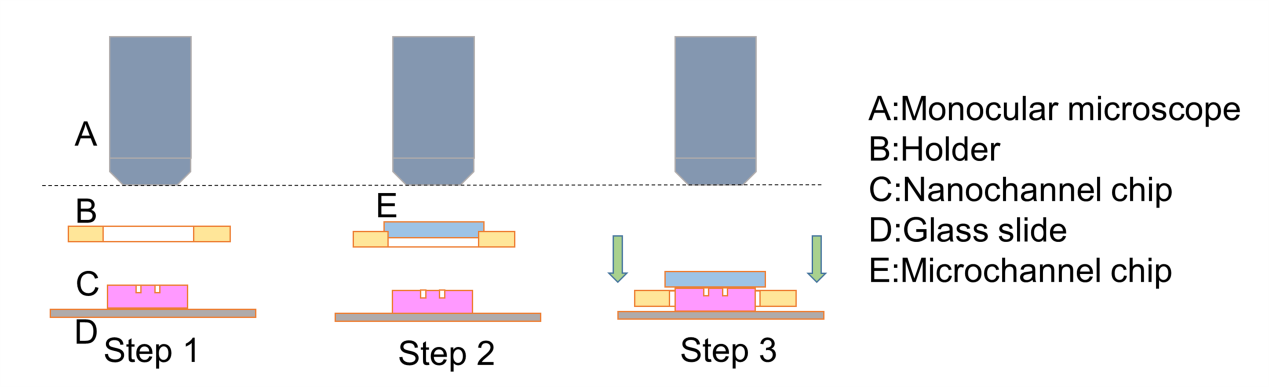


Fig. S2. Schematic illustrations of alignment procedures during bonding process.

**Rotary trajectory of piezoelectric actuator**





Fig. S3. Relationship between scratching circle diameter and driving voltage.

**Fabrication of nanochannel molds on PC sheet**

**
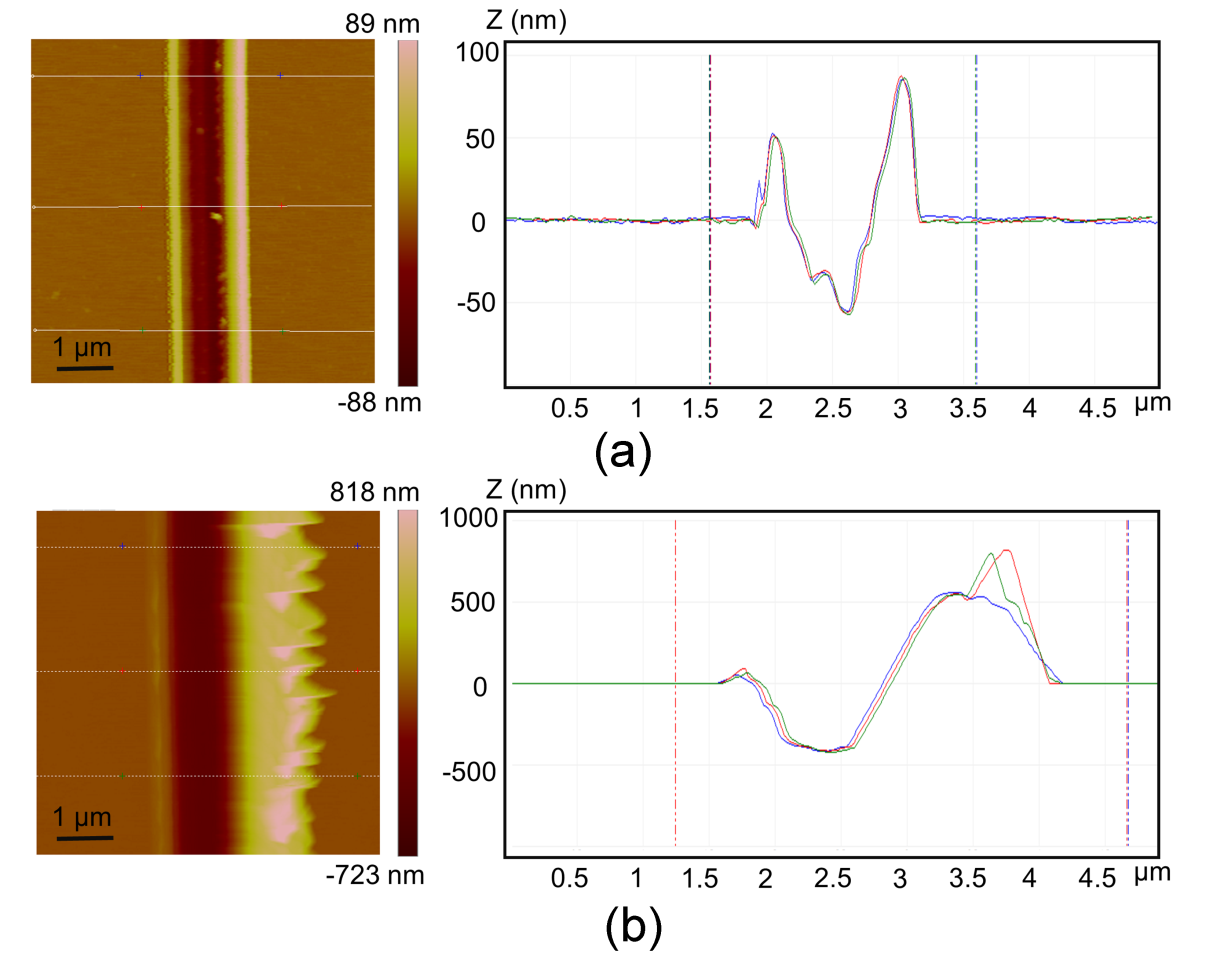
**

Fig. S4. Typical AFM images of the machined nanochannel with different machining parameters: (a) Nanomilling under a normal load of 17 μN, a frequency of 100 Hz, and a driving voltage of 90 V, (b) Nanomilling under a normal load of 25 μN, a frequency of 1500 Hz, and a driving voltage of 120 V.

**First transfer of nanochannel molds**

The changes in height and in width of the wall transferred from the fabricated “nanochannel I” are illustrated in Figs. S5(a) and S5(b), respectively, and the black dash line represents the original nanochannel size before transfer. Fig. S5(a) reveals that the heights of the wall transferred at different PDMS weight ratios were very close to each other, and the values were smaller than the original machining depth. It is observable from Fig. S5(b) that the widths of the wall were larger than the original machining size, moreover, the width of the wall transferred at a PDMS weight ratio of 5:1 was larger than the values at other ratios. Fig. S6 presents a typical AFM image of the wall fabricated from nanochannel I at a ratio of 5:1 during first transfer, where “W_f_” and “D_f_” are the width and the height of the wall, respectively, and this wall was called as “wall I.” Moreover, wall I manifested a better transfer quality, thus, the nanochannel shape was completely replicated to the PDMS chip.


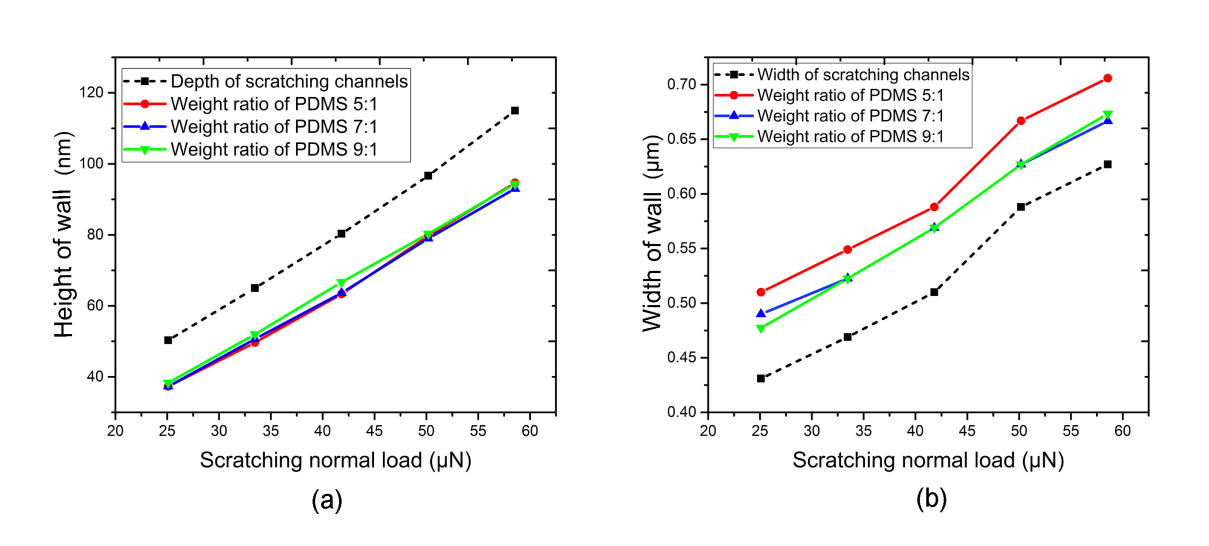


Fig. S5. Relationship between wall size and transfer parameters (various weight ratio of PDMS) during first transfer process, where the channel molds were fabricated with single scratching approach: (a) Wall height, (b) Wall width.

**
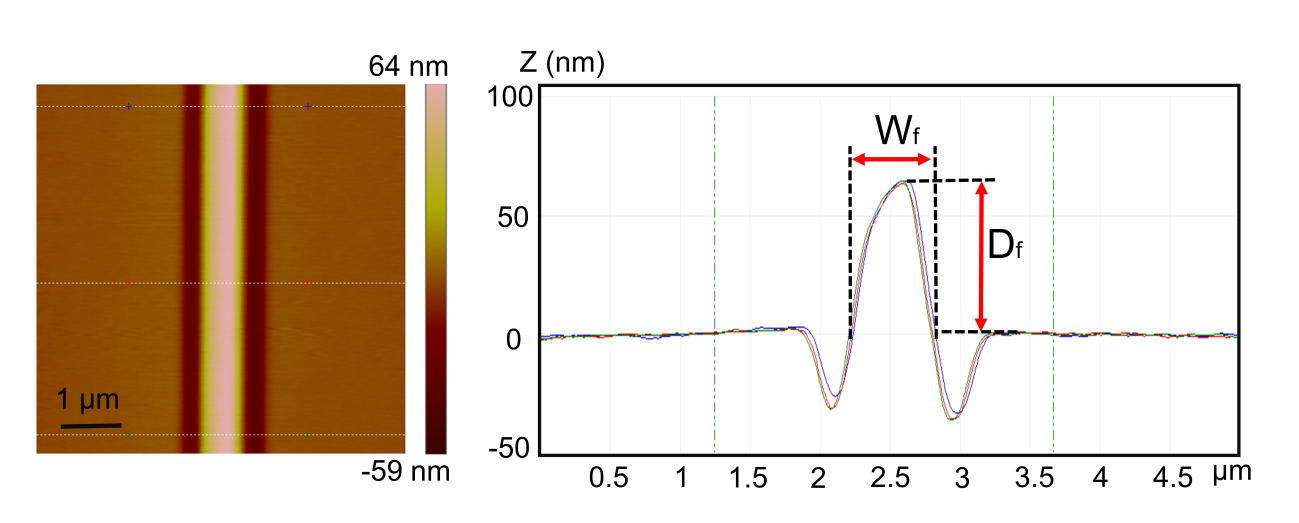
**

Fig. S6. Typical AFM image (left) and corresponding cross-section (right) of the wall obtained from nanochannel I at a PDMS weight ratio of 5:1 during first transfer.

Figs. S7(a) and S7(b) exhibit the changes in height and in weight of the wall under a normal load of 17 μN and a frequency of 100 Hz. It is evident that the heights of the wall at different PDMS weight ratios were approximately the same, and the values were smaller than the original machining size. Moreover, the widths of the wall were larger than the initial machining size, and the width at the ratio of 5:1 was found to be the largest. Fig. S8 displays a typical AFM image of the wall obtained from nanochannel II at a weight ratio of 5:1 during first transfer, and this wall was termed as “wall II”. Moreover, wall II manifested a better transfer quality, thus, the nanochannel shape was completely replicated to the PDMS chip.


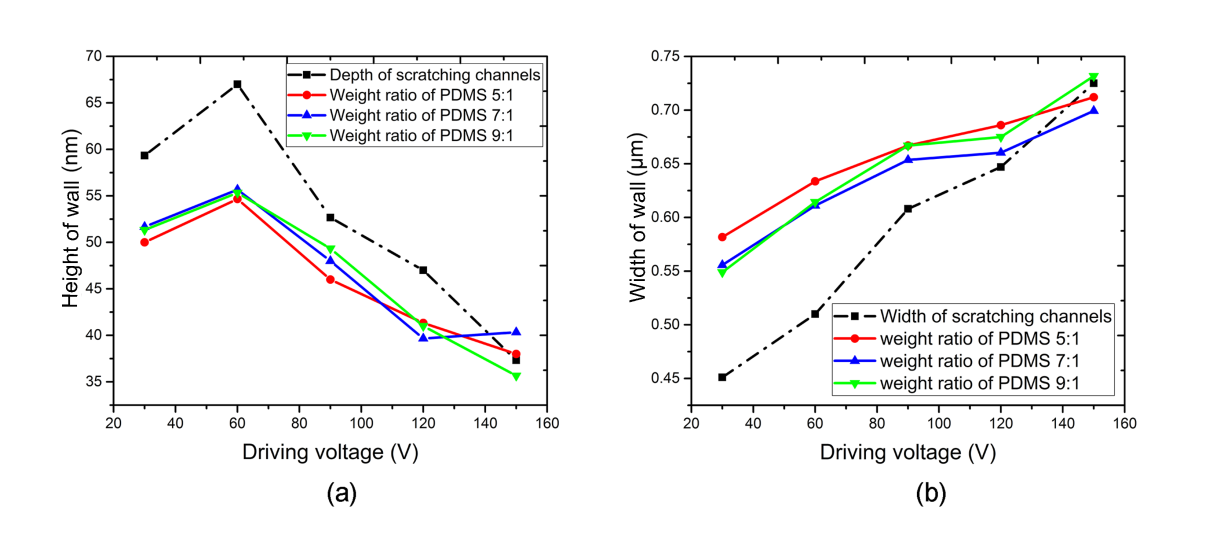


Fig. S7. Relationship between wall size and transfer parameters (various weight ratio of PDMS) during first transfer process, where the channel molds were fabricated with a normal load of 17 μN and a frequency of 100 Hz: (a) Wall height, (b) Wall width.

**
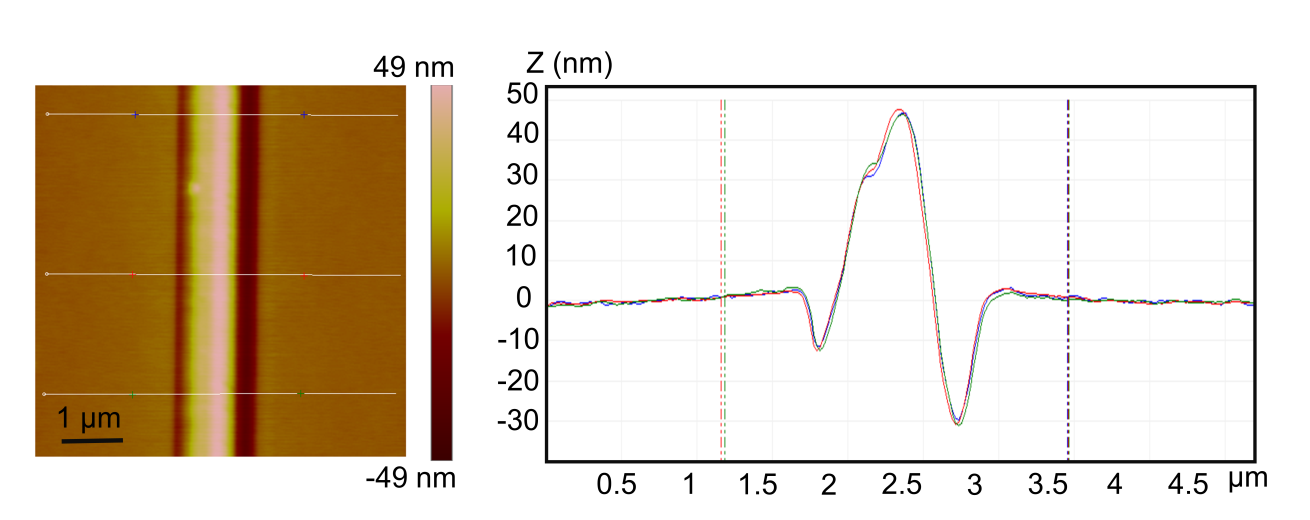
**

Fig. S8. Typical AFM image (left) and corresponding cross-section (right) of the wall obtained from nanochannel II at a weight ratio of 5:1 during first transfer.

**Second transfer of nanochannel molds**

Figs. S9(a) and S9(b) present the changes in depth and in width of the wall for different PDMS weight ratios. It is observable from Fig. S9(a) that the depths obtained at 8:1 and 9:1 were equal to each other and also were very close to the original machining size; however, the depth obtained at 10:1 was smaller than other two values. The changing trend of width in Fig. S9(b) was identical to the changes in depth; however, the deviation in width between 9:1 and 8:1 was larger than the deviation in depth. Fig. S10 displays a typical AFM image of the nanochannel (60 nm width and 500 nm width) obtained from wall I at a weight ratio of 10:1 during second transfer, and it was termed as “nanochannel A”. It is evident that the morphology of the nanochannel was successfully duplicated with a high quality.


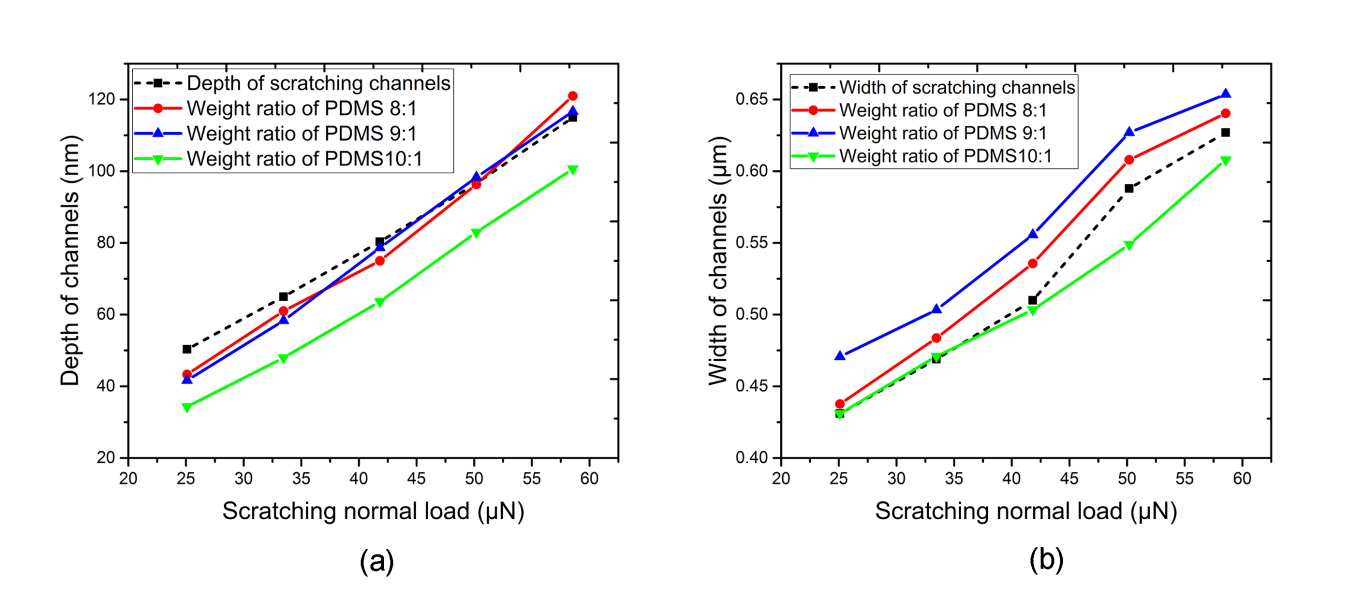


Fig. S9. Relationship between nanochannel size and transfer parameters (various weight ratio of PDMS) during second transfer, where the channel molds were fabricated with single scratching approach: (a) Nanochannel depth, (b) Nanochannel width.

**
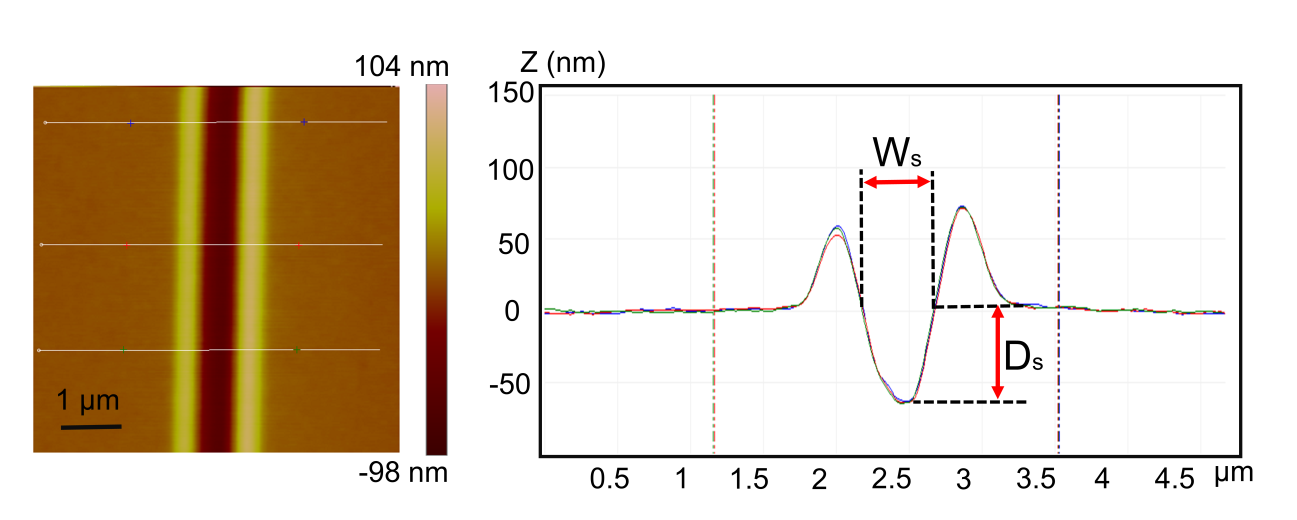
**

Fig. S10. Typical AFM image (left) and corresponding cross-section (right) of the nanochannel obtained from wall I at a weight ratio of 10:1 during second transfer.

Fig. S11 presents the relationship between nanochannel size obtained under a normal load of 17 μN and a frequency of 100 Hz and transfer parameters during second transfer. It is clear from Fig. S11(a) that the depths of the nanochannels were larger than the original machining size, moreover, the depth at 10:1 was found to be larger than other two ratios. Further, the widths of the wall were also larger than the original size, and the width at 10:1 was found to be the largest (Fig. S11(b)). Fig. S12 exhibits the typical AFM image of the nanochannel (80 nm height and 680 nm width) obtained from wall II at a weight ratio of 10:1, and it was called as “nanochannnel B”. The bottom structure of the original nanochannel was completely duplicated to nanochannel B.

**
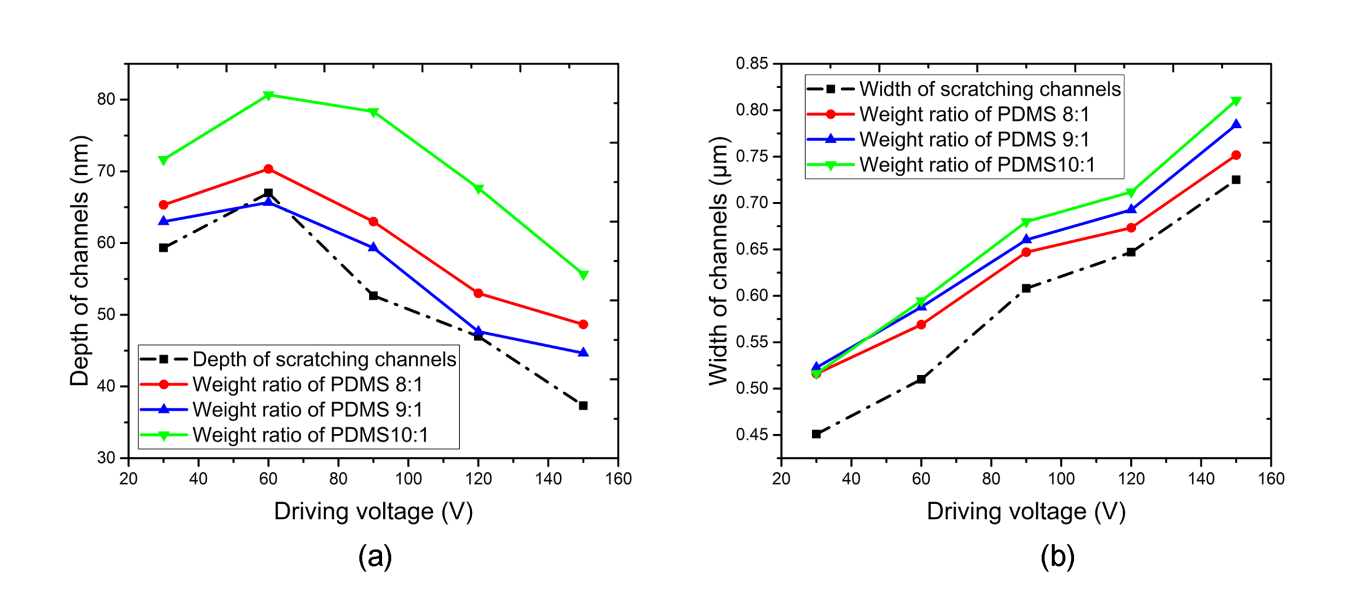
**

Fig. S11. Relationship between nanochannel size and transfer parameters (various weight ratio of PDMS) during second transfer, where the channel molds were fabricated with a normal load of 17 μN and a frequency of 100 Hz: (a) Nanochannel depth, (b) Nanochannel width.

**
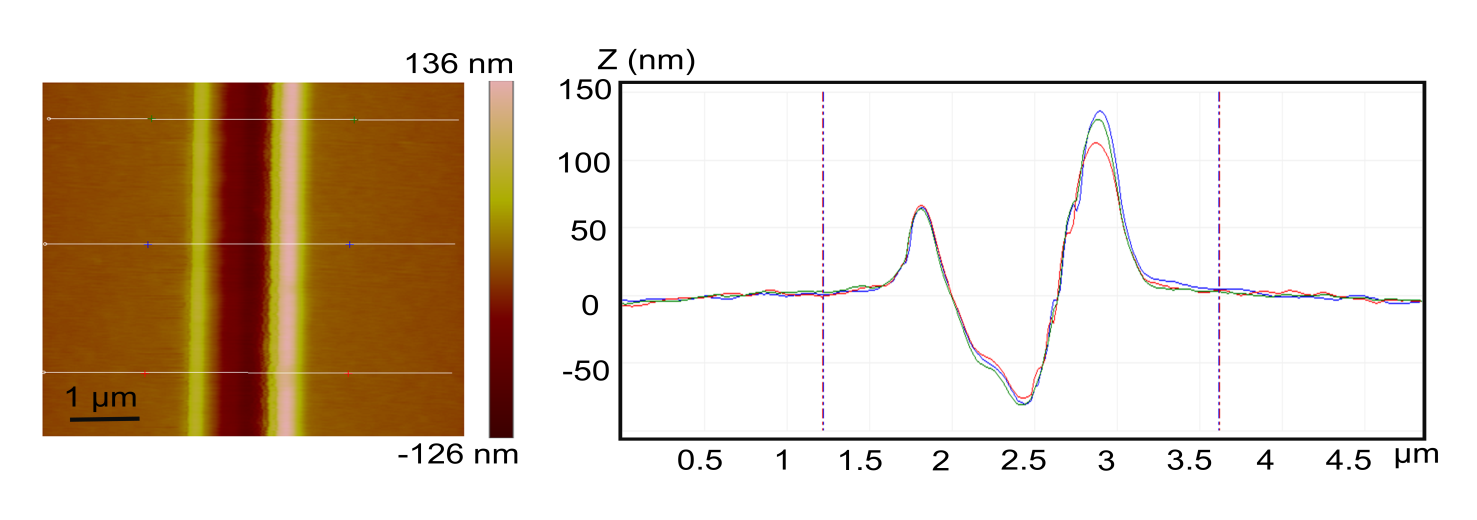
**

Fig. S12. Typical AFM image (left) and corresponding cross-section (right) of the nanochannel obtained from wall II at a weight ratio of 10:1 during second transfer.
