# Supplementary figures and images for: Fabrication of polydimethylsiloxane nanofluidic chips under AFM tip-based nanomilling process
Source: Nanoscale Res Lett. 2019 Apr 17;14:136. doi: 10.1186/s11671-019-2962-6 (PMC6470239; doi:10.1186/s11671-019-2962-6)

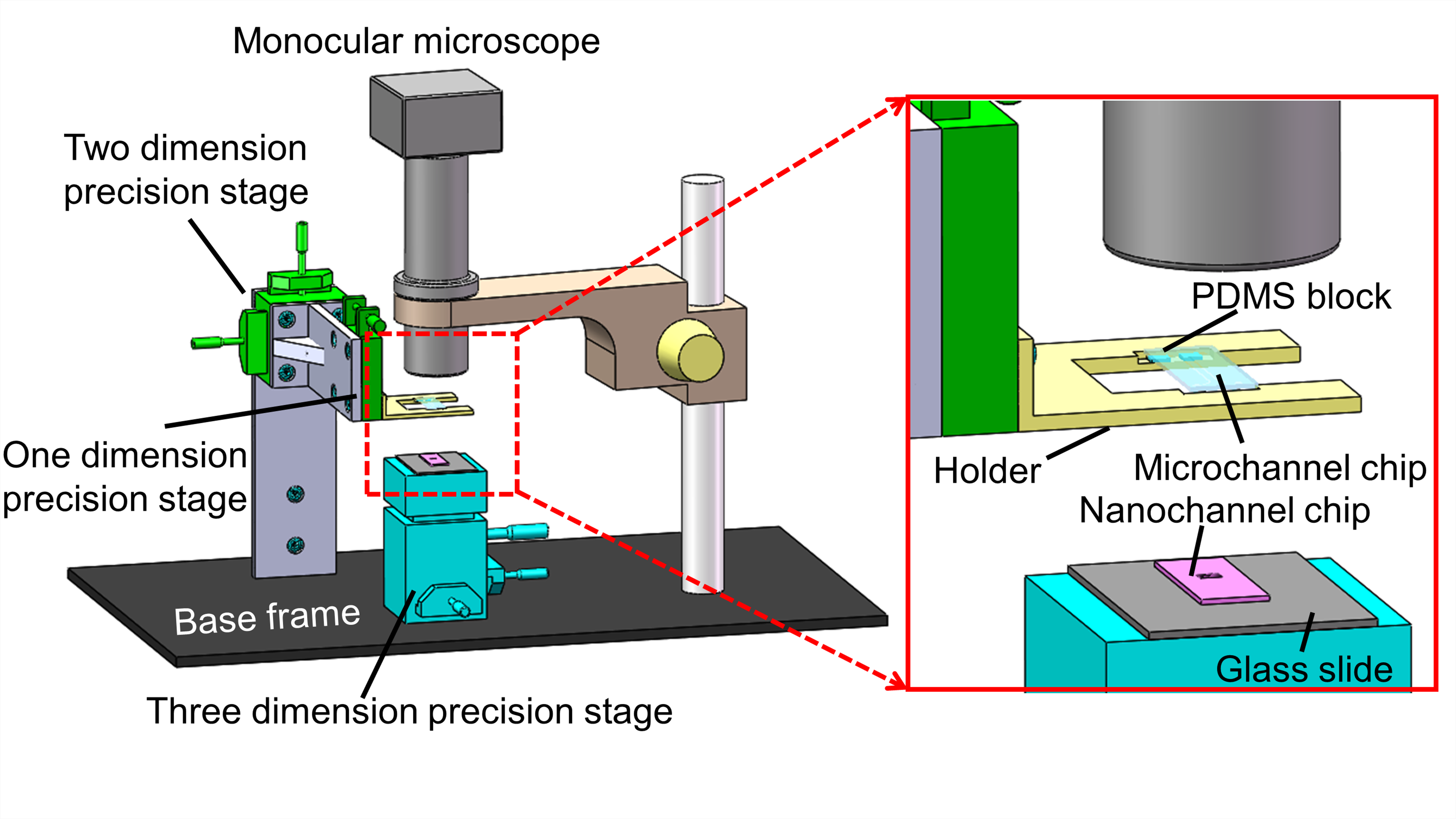

Supplement: Supplementary file 1 — Figure S1. Schematic diagram of homemade alignment system. Figure S2. Schematic illustrations of alignment procedures during bonding process. Figure S3. Relationship between scratching circle diameter and driving voltage. Figure S4. Typical AFM images of the machined nanochannel with different machining parameters. Figure S5. Relationship between wall size and transfer parameters (various weight ratio of PDMS) during first transfer process, where the channel molds were fabricated with single scratching approach: (a) Wall height, (b) Wall width. Figure S6. Typical AFM image (left) and corresponding cross-section (right) of the wall obtained from nanochannel I at a PDMS weight ratio of 5:1 during first transfer. Figure S7. Relationship between wall size and transfer parameters (various weight ratio of PDMS) during first transfer process, where the channel molds were fabricated with a normal load of 17 μN and a frequency of 100 Hz: (a) Wall height, (b) Wall width. Figure S8. Typical AFM image (left) and corresponding cross-section (right) of the wall obtained from nanochannel II at a weight ratio of 5:1 during first transfer. Figure S9. Relationship between nanochannel size and transfer parameters (various weight ratio of PDMS) during second transfer, where the channel molds were fabricated with single scratching approach: (a) Nanochannel depth, (b) Nanochannel width. Figure S10. Typical AFM image (left) and corresponding cross-section (right) of the nanochannel obtained from wall I at a weight ratio of 10:1 during second transfer. Figure S11. Relationship between nanochannel size and transfer parameters (various weight ratio of PDMS) during second transfer, where the channel molds were fabricated with a normal load of 17 μN and a frequency of 100 Hz: (a) Nanochannel depth, (b) Nanochannel width. Figure S12. Typical AFM image (left) and corresponding cross-section (right) of the nanochannel obtained from wall II at a weight ratio of 10:1 during second transfer. (ZIP 16 [file 11671_2019_2962_MOESM1_ESM.zip › Fig. S1.tif]

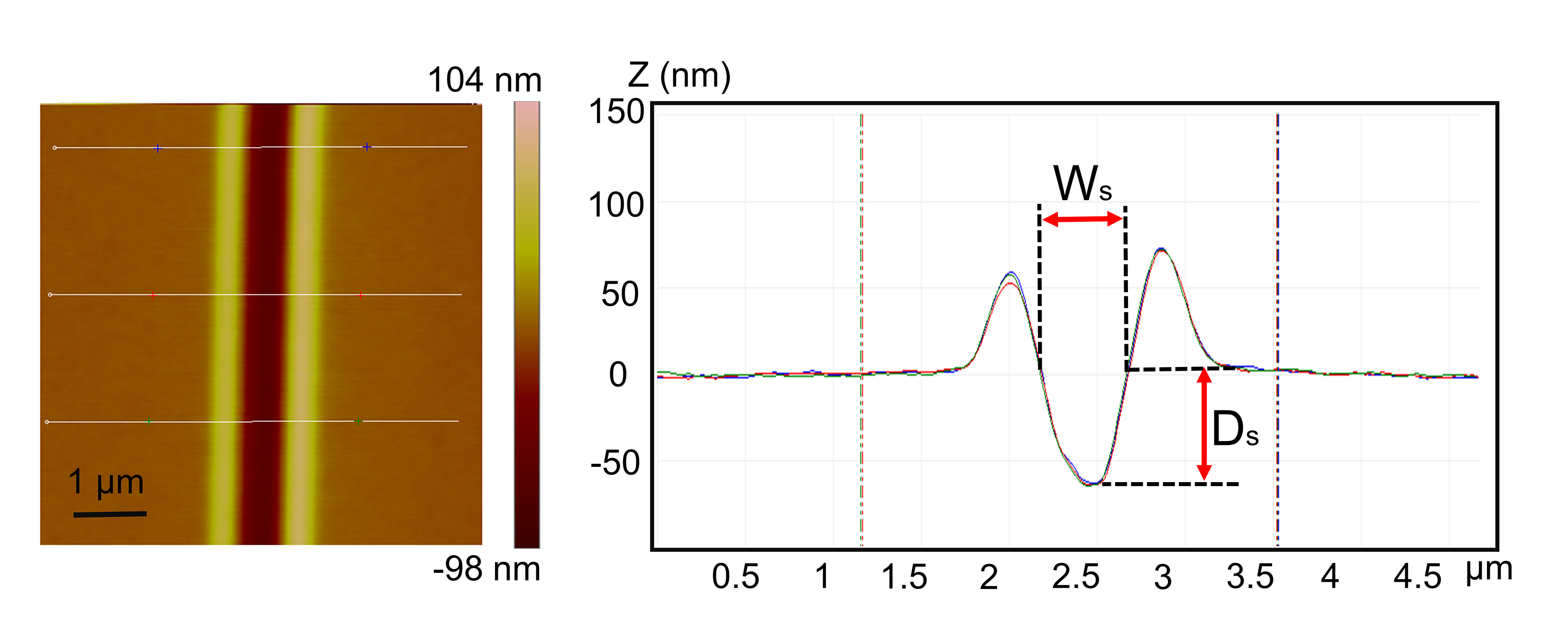

Supplement: Supplementary file 1 — Figure S1. Schematic diagram of homemade alignment system. Figure S2. Schematic illustrations of alignment procedures during bonding process. Figure S3. Relationship between scratching circle diameter and driving voltage. Figure S4. Typical AFM images of the machined nanochannel with different machining parameters. Figure S5. Relationship between wall size and transfer parameters (various weight ratio of PDMS) during first transfer process, where the channel molds were fabricated with single scratching approach: (a) Wall height, (b) Wall width. Figure S6. Typical AFM image (left) and corresponding cross-section (right) of the wall obtained from nanochannel I at a PDMS weight ratio of 5:1 during first transfer. Figure S7. Relationship between wall size and transfer parameters (various weight ratio of PDMS) during first transfer process, where the channel molds were fabricated with a normal load of 17 μN and a frequency of 100 Hz: (a) Wall height, (b) Wall width. Figure S8. Typical AFM image (left) and corresponding cross-section (right) of the wall obtained from nanochannel II at a weight ratio of 5:1 during first transfer. Figure S9. Relationship between nanochannel size and transfer parameters (various weight ratio of PDMS) during second transfer, where the channel molds were fabricated with single scratching approach: (a) Nanochannel depth, (b) Nanochannel width. Figure S10. Typical AFM image (left) and corresponding cross-section (right) of the nanochannel obtained from wall I at a weight ratio of 10:1 during second transfer. Figure S11. Relationship between nanochannel size and transfer parameters (various weight ratio of PDMS) during second transfer, where the channel molds were fabricated with a normal load of 17 μN and a frequency of 100 Hz: (a) Nanochannel depth, (b) Nanochannel width. Figure S12. Typical AFM image (left) and corresponding cross-section (right) of the nanochannel obtained from wall II at a weight ratio of 10:1 during second transfer. (ZIP 16 [file 11671_2019_2962_MOESM1_ESM.zip › Fig. s10.tif]

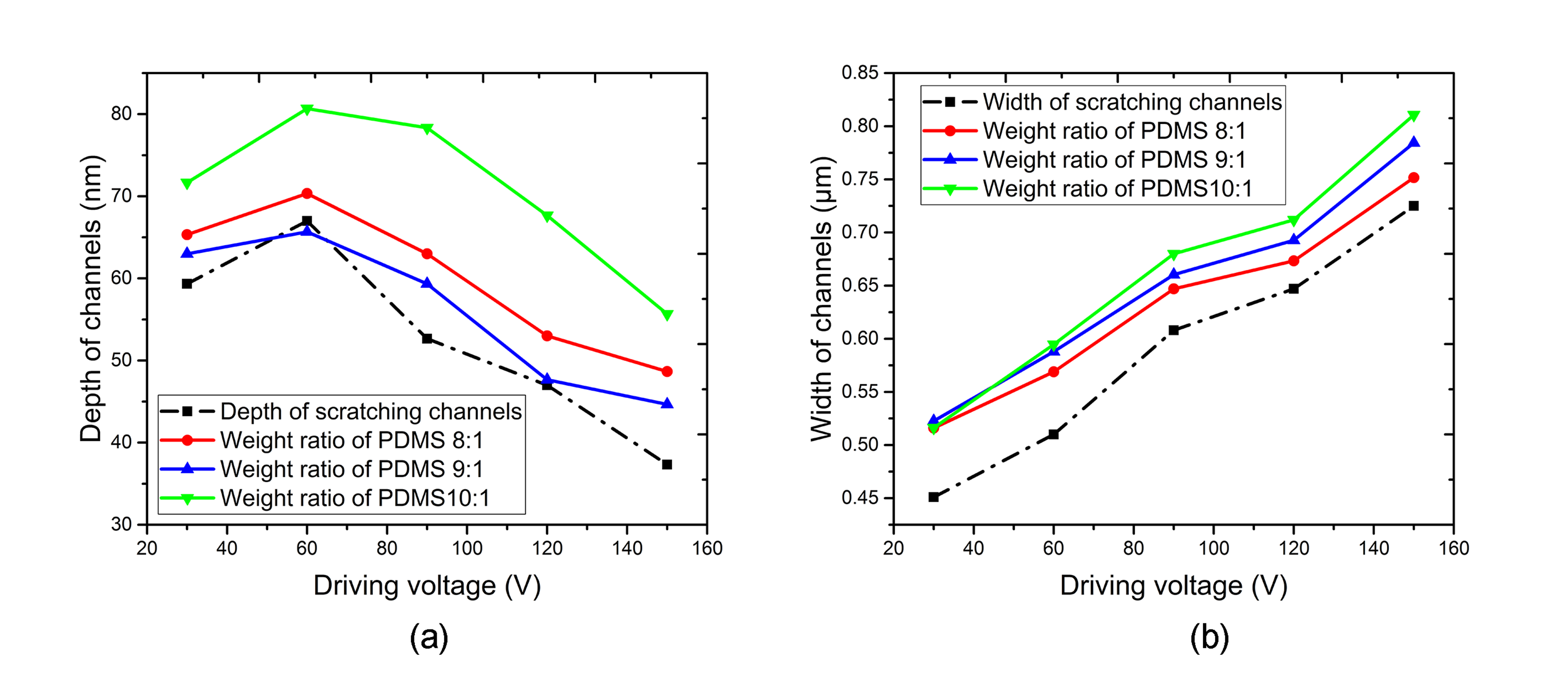

Supplement: Supplementary file 1 — Figure S1. Schematic diagram of homemade alignment system. Figure S2. Schematic illustrations of alignment procedures during bonding process. Figure S3. Relationship between scratching circle diameter and driving voltage. Figure S4. Typical AFM images of the machined nanochannel with different machining parameters. Figure S5. Relationship between wall size and transfer parameters (various weight ratio of PDMS) during first transfer process, where the channel molds were fabricated with single scratching approach: (a) Wall height, (b) Wall width. Figure S6. Typical AFM image (left) and corresponding cross-section (right) of the wall obtained from nanochannel I at a PDMS weight ratio of 5:1 during first transfer. Figure S7. Relationship between wall size and transfer parameters (various weight ratio of PDMS) during first transfer process, where the channel molds were fabricated with a normal load of 17 μN and a frequency of 100 Hz: (a) Wall height, (b) Wall width. Figure S8. Typical AFM image (left) and corresponding cross-section (right) of the wall obtained from nanochannel II at a weight ratio of 5:1 during first transfer. Figure S9. Relationship between nanochannel size and transfer parameters (various weight ratio of PDMS) during second transfer, where the channel molds were fabricated with single scratching approach: (a) Nanochannel depth, (b) Nanochannel width. Figure S10. Typical AFM image (left) and corresponding cross-section (right) of the nanochannel obtained from wall I at a weight ratio of 10:1 during second transfer. Figure S11. Relationship between nanochannel size and transfer parameters (various weight ratio of PDMS) during second transfer, where the channel molds were fabricated with a normal load of 17 μN and a frequency of 100 Hz: (a) Nanochannel depth, (b) Nanochannel width. Figure S12. Typical AFM image (left) and corresponding cross-section (right) of the nanochannel obtained from wall II at a weight ratio of 10:1 during second transfer. (ZIP 16 [file 11671_2019_2962_MOESM1_ESM.zip › Fig. s11.tif]

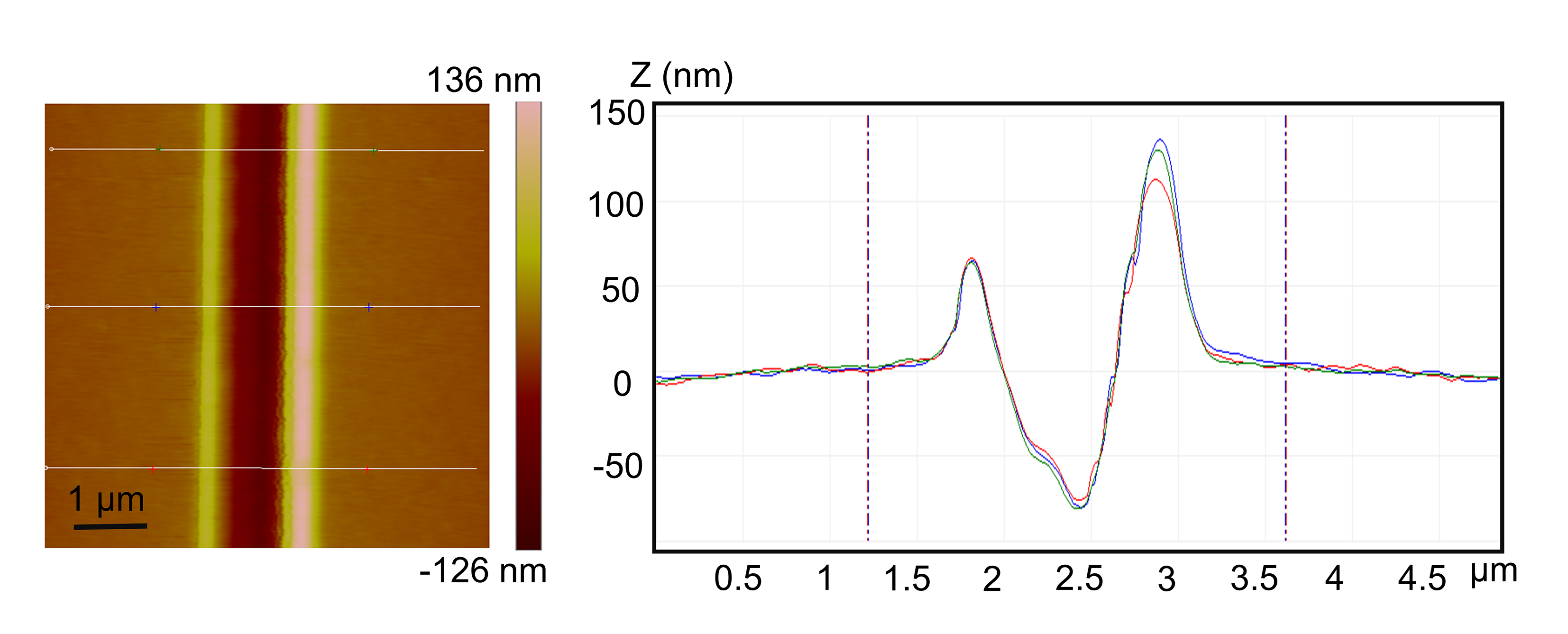

Supplement: Supplementary file 1 — Figure S1. Schematic diagram of homemade alignment system. Figure S2. Schematic illustrations of alignment procedures during bonding process. Figure S3. Relationship between scratching circle diameter and driving voltage. Figure S4. Typical AFM images of the machined nanochannel with different machining parameters. Figure S5. Relationship between wall size and transfer parameters (various weight ratio of PDMS) during first transfer process, where the channel molds were fabricated with single scratching approach: (a) Wall height, (b) Wall width. Figure S6. Typical AFM image (left) and corresponding cross-section (right) of the wall obtained from nanochannel I at a PDMS weight ratio of 5:1 during first transfer. Figure S7. Relationship between wall size and transfer parameters (various weight ratio of PDMS) during first transfer process, where the channel molds were fabricated with a normal load of 17 μN and a frequency of 100 Hz: (a) Wall height, (b) Wall width. Figure S8. Typical AFM image (left) and corresponding cross-section (right) of the wall obtained from nanochannel II at a weight ratio of 5:1 during first transfer. Figure S9. Relationship between nanochannel size and transfer parameters (various weight ratio of PDMS) during second transfer, where the channel molds were fabricated with single scratching approach: (a) Nanochannel depth, (b) Nanochannel width. Figure S10. Typical AFM image (left) and corresponding cross-section (right) of the nanochannel obtained from wall I at a weight ratio of 10:1 during second transfer. Figure S11. Relationship between nanochannel size and transfer parameters (various weight ratio of PDMS) during second transfer, where the channel molds were fabricated with a normal load of 17 μN and a frequency of 100 Hz: (a) Nanochannel depth, (b) Nanochannel width. Figure S12. Typical AFM image (left) and corresponding cross-section (right) of the nanochannel obtained from wall II at a weight ratio of 10:1 during second transfer. (ZIP 16 [file 11671_2019_2962_MOESM1_ESM.zip › Fig. s12.tif]

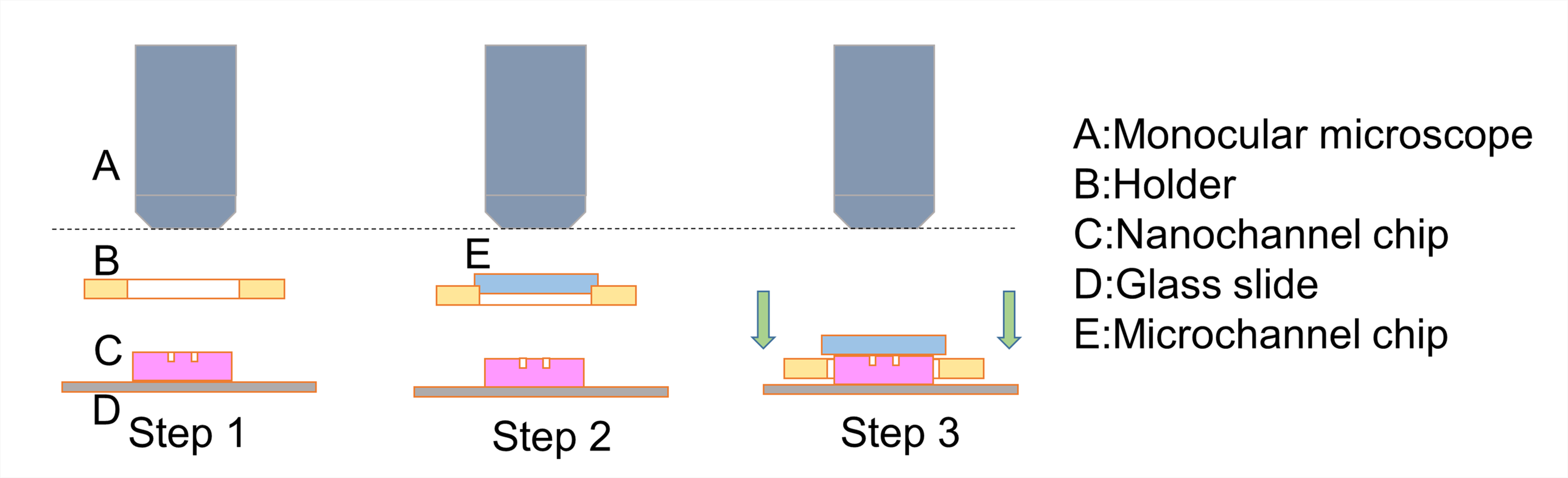

Supplement: Supplementary file 1 — Figure S1. Schematic diagram of homemade alignment system. Figure S2. Schematic illustrations of alignment procedures during bonding process. Figure S3. Relationship between scratching circle diameter and driving voltage. Figure S4. Typical AFM images of the machined nanochannel with different machining parameters. Figure S5. Relationship between wall size and transfer parameters (various weight ratio of PDMS) during first transfer process, where the channel molds were fabricated with single scratching approach: (a) Wall height, (b) Wall width. Figure S6. Typical AFM image (left) and corresponding cross-section (right) of the wall obtained from nanochannel I at a PDMS weight ratio of 5:1 during first transfer. Figure S7. Relationship between wall size and transfer parameters (various weight ratio of PDMS) during first transfer process, where the channel molds were fabricated with a normal load of 17 μN and a frequency of 100 Hz: (a) Wall height, (b) Wall width. Figure S8. Typical AFM image (left) and corresponding cross-section (right) of the wall obtained from nanochannel II at a weight ratio of 5:1 during first transfer. Figure S9. Relationship between nanochannel size and transfer parameters (various weight ratio of PDMS) during second transfer, where the channel molds were fabricated with single scratching approach: (a) Nanochannel depth, (b) Nanochannel width. Figure S10. Typical AFM image (left) and corresponding cross-section (right) of the nanochannel obtained from wall I at a weight ratio of 10:1 during second transfer. Figure S11. Relationship between nanochannel size and transfer parameters (various weight ratio of PDMS) during second transfer, where the channel molds were fabricated with a normal load of 17 μN and a frequency of 100 Hz: (a) Nanochannel depth, (b) Nanochannel width. Figure S12. Typical AFM image (left) and corresponding cross-section (right) of the nanochannel obtained from wall II at a weight ratio of 10:1 during second transfer. (ZIP 16 [file 11671_2019_2962_MOESM1_ESM.zip › Fig. S2.tif]

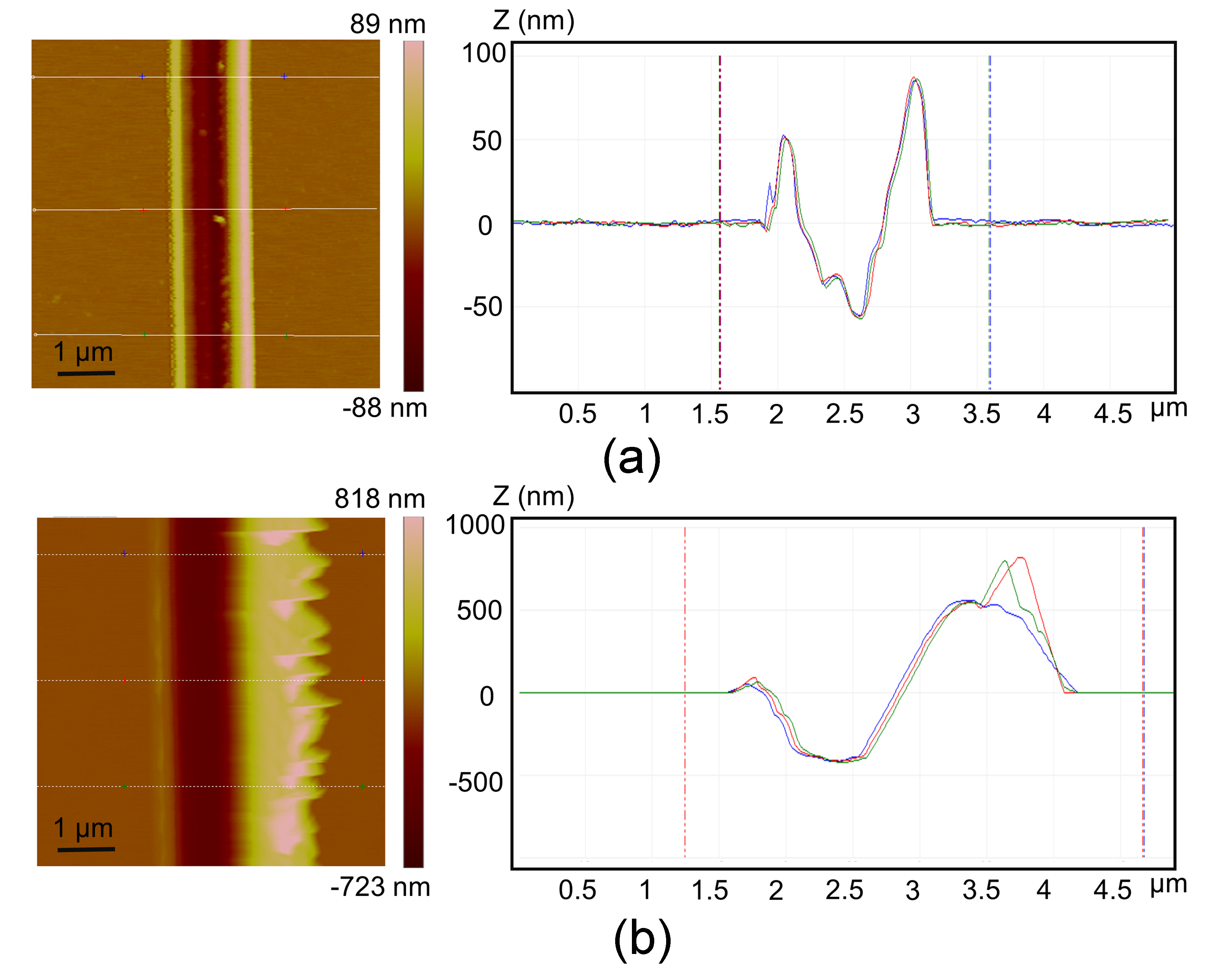

Supplement: Supplementary file 1 — Figure S1. Schematic diagram of homemade alignment system. Figure S2. Schematic illustrations of alignment procedures during bonding process. Figure S3. Relationship between scratching circle diameter and driving voltage. Figure S4. Typical AFM images of the machined nanochannel with different machining parameters. Figure S5. Relationship between wall size and transfer parameters (various weight ratio of PDMS) during first transfer process, where the channel molds were fabricated with single scratching approach: (a) Wall height, (b) Wall width. Figure S6. Typical AFM image (left) and corresponding cross-section (right) of the wall obtained from nanochannel I at a PDMS weight ratio of 5:1 during first transfer. Figure S7. Relationship between wall size and transfer parameters (various weight ratio of PDMS) during first transfer process, where the channel molds were fabricated with a normal load of 17 μN and a frequency of 100 Hz: (a) Wall height, (b) Wall width. Figure S8. Typical AFM image (left) and corresponding cross-section (right) of the wall obtained from nanochannel II at a weight ratio of 5:1 during first transfer. Figure S9. Relationship between nanochannel size and transfer parameters (various weight ratio of PDMS) during second transfer, where the channel molds were fabricated with single scratching approach: (a) Nanochannel depth, (b) Nanochannel width. Figure S10. Typical AFM image (left) and corresponding cross-section (right) of the nanochannel obtained from wall I at a weight ratio of 10:1 during second transfer. Figure S11. Relationship between nanochannel size and transfer parameters (various weight ratio of PDMS) during second transfer, where the channel molds were fabricated with a normal load of 17 μN and a frequency of 100 Hz: (a) Nanochannel depth, (b) Nanochannel width. Figure S12. Typical AFM image (left) and corresponding cross-section (right) of the nanochannel obtained from wall II at a weight ratio of 10:1 during second transfer. (ZIP 16 [file 11671_2019_2962_MOESM1_ESM.zip › Fig. s4.tif]

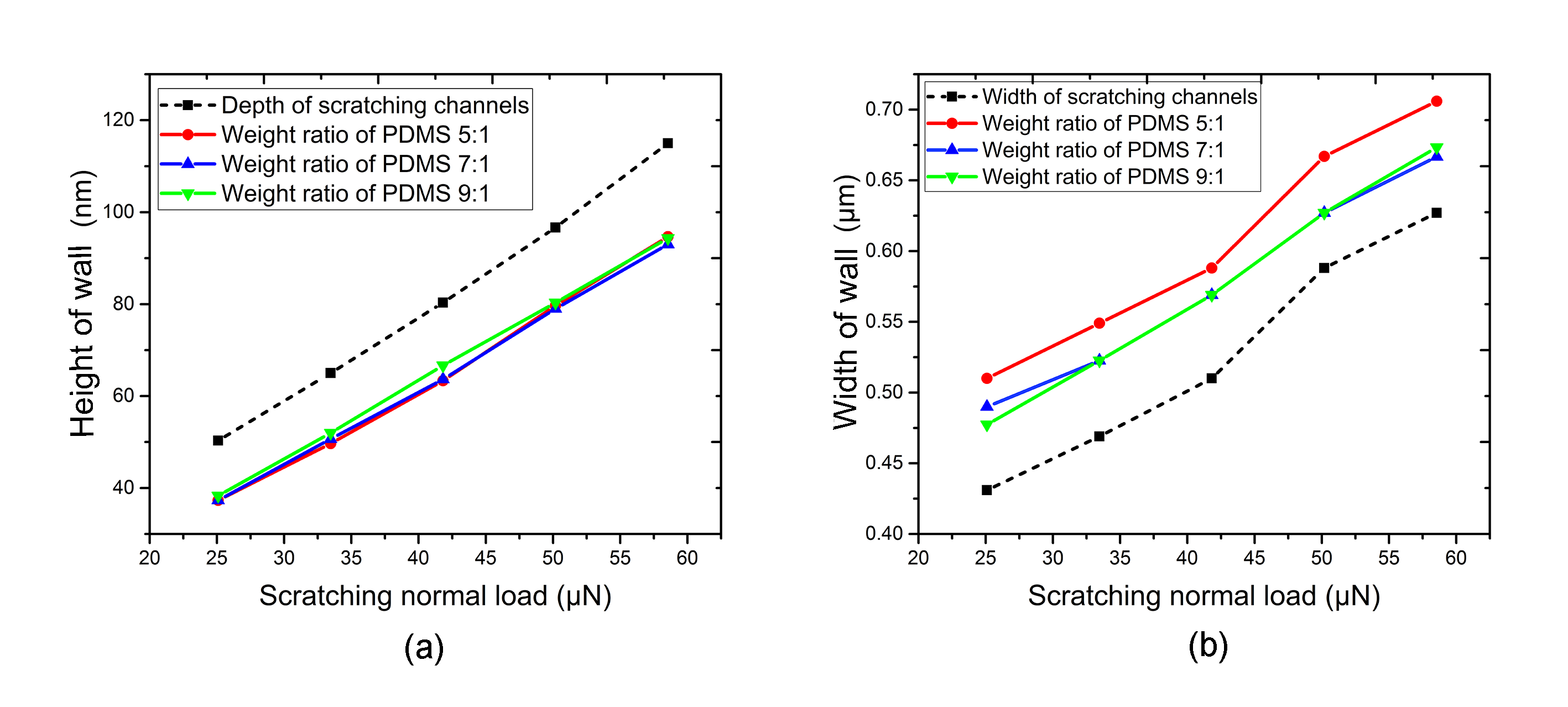

Supplement: Supplementary file 1 — Figure S1. Schematic diagram of homemade alignment system. Figure S2. Schematic illustrations of alignment procedures during bonding process. Figure S3. Relationship between scratching circle diameter and driving voltage. Figure S4. Typical AFM images of the machined nanochannel with different machining parameters. Figure S5. Relationship between wall size and transfer parameters (various weight ratio of PDMS) during first transfer process, where the channel molds were fabricated with single scratching approach: (a) Wall height, (b) Wall width. Figure S6. Typical AFM image (left) and corresponding cross-section (right) of the wall obtained from nanochannel I at a PDMS weight ratio of 5:1 during first transfer. Figure S7. Relationship between wall size and transfer parameters (various weight ratio of PDMS) during first transfer process, where the channel molds were fabricated with a normal load of 17 μN and a frequency of 100 Hz: (a) Wall height, (b) Wall width. Figure S8. Typical AFM image (left) and corresponding cross-section (right) of the wall obtained from nanochannel II at a weight ratio of 5:1 during first transfer. Figure S9. Relationship between nanochannel size and transfer parameters (various weight ratio of PDMS) during second transfer, where the channel molds were fabricated with single scratching approach: (a) Nanochannel depth, (b) Nanochannel width. Figure S10. Typical AFM image (left) and corresponding cross-section (right) of the nanochannel obtained from wall I at a weight ratio of 10:1 during second transfer. Figure S11. Relationship between nanochannel size and transfer parameters (various weight ratio of PDMS) during second transfer, where the channel molds were fabricated with a normal load of 17 μN and a frequency of 100 Hz: (a) Nanochannel depth, (b) Nanochannel width. Figure S12. Typical AFM image (left) and corresponding cross-section (right) of the nanochannel obtained from wall II at a weight ratio of 10:1 during second transfer. (ZIP 16 [file 11671_2019_2962_MOESM1_ESM.zip › Fig. s5.tif]

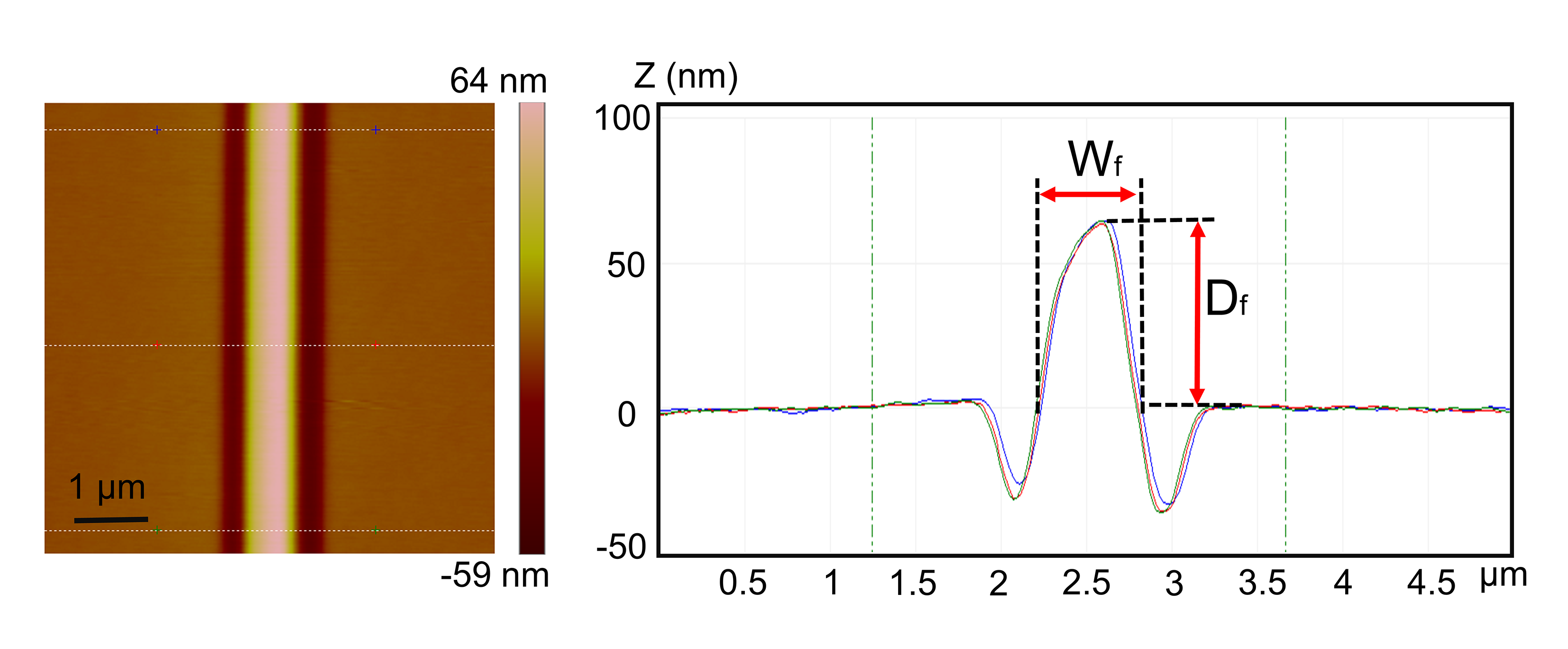

Supplement: Supplementary file 1 — Figure S1. Schematic diagram of homemade alignment system. Figure S2. Schematic illustrations of alignment procedures during bonding process. Figure S3. Relationship between scratching circle diameter and driving voltage. Figure S4. Typical AFM images of the machined nanochannel with different machining parameters. Figure S5. Relationship between wall size and transfer parameters (various weight ratio of PDMS) during first transfer process, where the channel molds were fabricated with single scratching approach: (a) Wall height, (b) Wall width. Figure S6. Typical AFM image (left) and corresponding cross-section (right) of the wall obtained from nanochannel I at a PDMS weight ratio of 5:1 during first transfer. Figure S7. Relationship between wall size and transfer parameters (various weight ratio of PDMS) during first transfer process, where the channel molds were fabricated with a normal load of 17 μN and a frequency of 100 Hz: (a) Wall height, (b) Wall width. Figure S8. Typical AFM image (left) and corresponding cross-section (right) of the wall obtained from nanochannel II at a weight ratio of 5:1 during first transfer. Figure S9. Relationship between nanochannel size and transfer parameters (various weight ratio of PDMS) during second transfer, where the channel molds were fabricated with single scratching approach: (a) Nanochannel depth, (b) Nanochannel width. Figure S10. Typical AFM image (left) and corresponding cross-section (right) of the nanochannel obtained from wall I at a weight ratio of 10:1 during second transfer. Figure S11. Relationship between nanochannel size and transfer parameters (various weight ratio of PDMS) during second transfer, where the channel molds were fabricated with a normal load of 17 μN and a frequency of 100 Hz: (a) Nanochannel depth, (b) Nanochannel width. Figure S12. Typical AFM image (left) and corresponding cross-section (right) of the nanochannel obtained from wall II at a weight ratio of 10:1 during second transfer. (ZIP 16 [file 11671_2019_2962_MOESM1_ESM.zip › Fig. s6.tif]

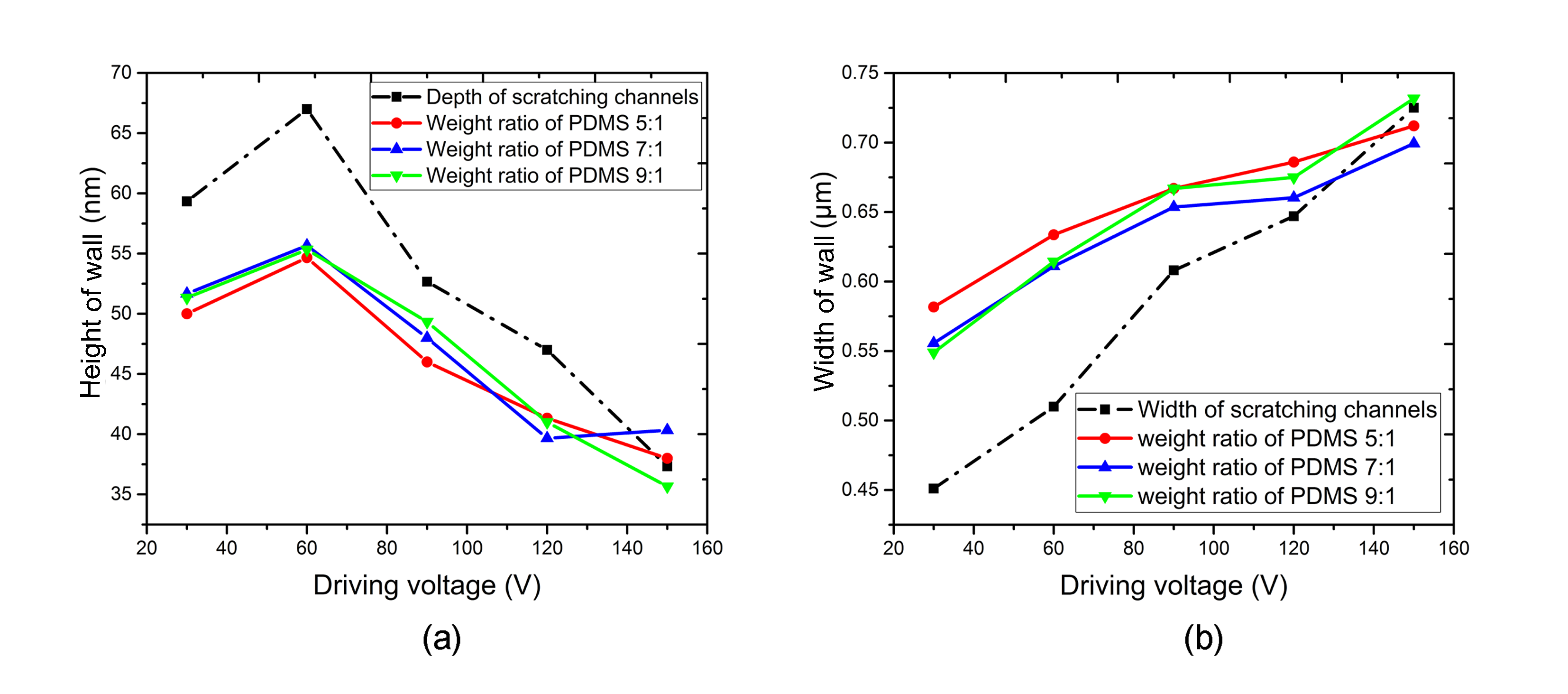

Supplement: Supplementary file 1 — Figure S1. Schematic diagram of homemade alignment system. Figure S2. Schematic illustrations of alignment procedures during bonding process. Figure S3. Relationship between scratching circle diameter and driving voltage. Figure S4. Typical AFM images of the machined nanochannel with different machining parameters. Figure S5. Relationship between wall size and transfer parameters (various weight ratio of PDMS) during first transfer process, where the channel molds were fabricated with single scratching approach: (a) Wall height, (b) Wall width. Figure S6. Typical AFM image (left) and corresponding cross-section (right) of the wall obtained from nanochannel I at a PDMS weight ratio of 5:1 during first transfer. Figure S7. Relationship between wall size and transfer parameters (various weight ratio of PDMS) during first transfer process, where the channel molds were fabricated with a normal load of 17 μN and a frequency of 100 Hz: (a) Wall height, (b) Wall width. Figure S8. Typical AFM image (left) and corresponding cross-section (right) of the wall obtained from nanochannel II at a weight ratio of 5:1 during first transfer. Figure S9. Relationship between nanochannel size and transfer parameters (various weight ratio of PDMS) during second transfer, where the channel molds were fabricated with single scratching approach: (a) Nanochannel depth, (b) Nanochannel width. Figure S10. Typical AFM image (left) and corresponding cross-section (right) of the nanochannel obtained from wall I at a weight ratio of 10:1 during second transfer. Figure S11. Relationship between nanochannel size and transfer parameters (various weight ratio of PDMS) during second transfer, where the channel molds were fabricated with a normal load of 17 μN and a frequency of 100 Hz: (a) Nanochannel depth, (b) Nanochannel width. Figure S12. Typical AFM image (left) and corresponding cross-section (right) of the nanochannel obtained from wall II at a weight ratio of 10:1 during second transfer. (ZIP 16 [file 11671_2019_2962_MOESM1_ESM.zip › Fig. s7.tif]

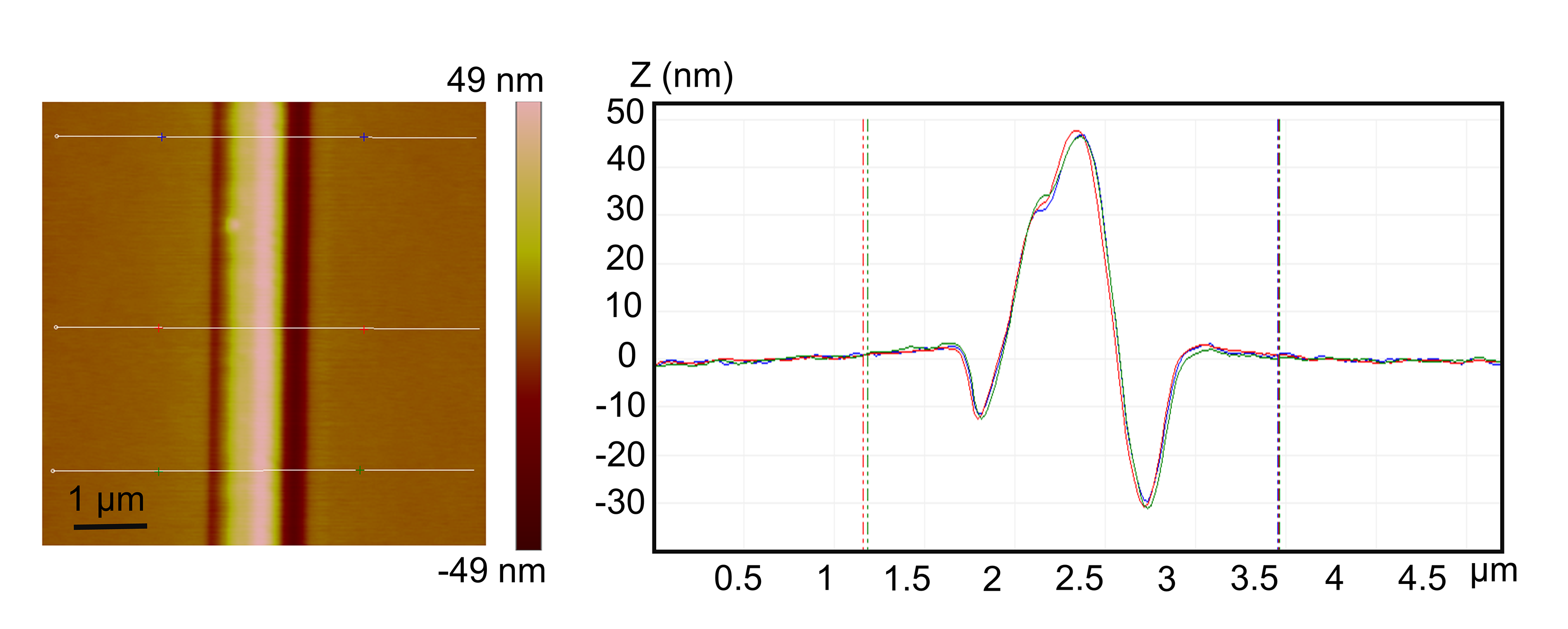

Supplement: Supplementary file 1 — Figure S1. Schematic diagram of homemade alignment system. Figure S2. Schematic illustrations of alignment procedures during bonding process. Figure S3. Relationship between scratching circle diameter and driving voltage. Figure S4. Typical AFM images of the machined nanochannel with different machining parameters. Figure S5. Relationship between wall size and transfer parameters (various weight ratio of PDMS) during first transfer process, where the channel molds were fabricated with single scratching approach: (a) Wall height, (b) Wall width. Figure S6. Typical AFM image (left) and corresponding cross-section (right) of the wall obtained from nanochannel I at a PDMS weight ratio of 5:1 during first transfer. Figure S7. Relationship between wall size and transfer parameters (various weight ratio of PDMS) during first transfer process, where the channel molds were fabricated with a normal load of 17 μN and a frequency of 100 Hz: (a) Wall height, (b) Wall width. Figure S8. Typical AFM image (left) and corresponding cross-section (right) of the wall obtained from nanochannel II at a weight ratio of 5:1 during first transfer. Figure S9. Relationship between nanochannel size and transfer parameters (various weight ratio of PDMS) during second transfer, where the channel molds were fabricated with single scratching approach: (a) Nanochannel depth, (b) Nanochannel width. Figure S10. Typical AFM image (left) and corresponding cross-section (right) of the nanochannel obtained from wall I at a weight ratio of 10:1 during second transfer. Figure S11. Relationship between nanochannel size and transfer parameters (various weight ratio of PDMS) during second transfer, where the channel molds were fabricated with a normal load of 17 μN and a frequency of 100 Hz: (a) Nanochannel depth, (b) Nanochannel width. Figure S12. Typical AFM image (left) and corresponding cross-section (right) of the nanochannel obtained from wall II at a weight ratio of 10:1 during second transfer. (ZIP 16 [file 11671_2019_2962_MOESM1_ESM.zip › Fig. s8.tif]

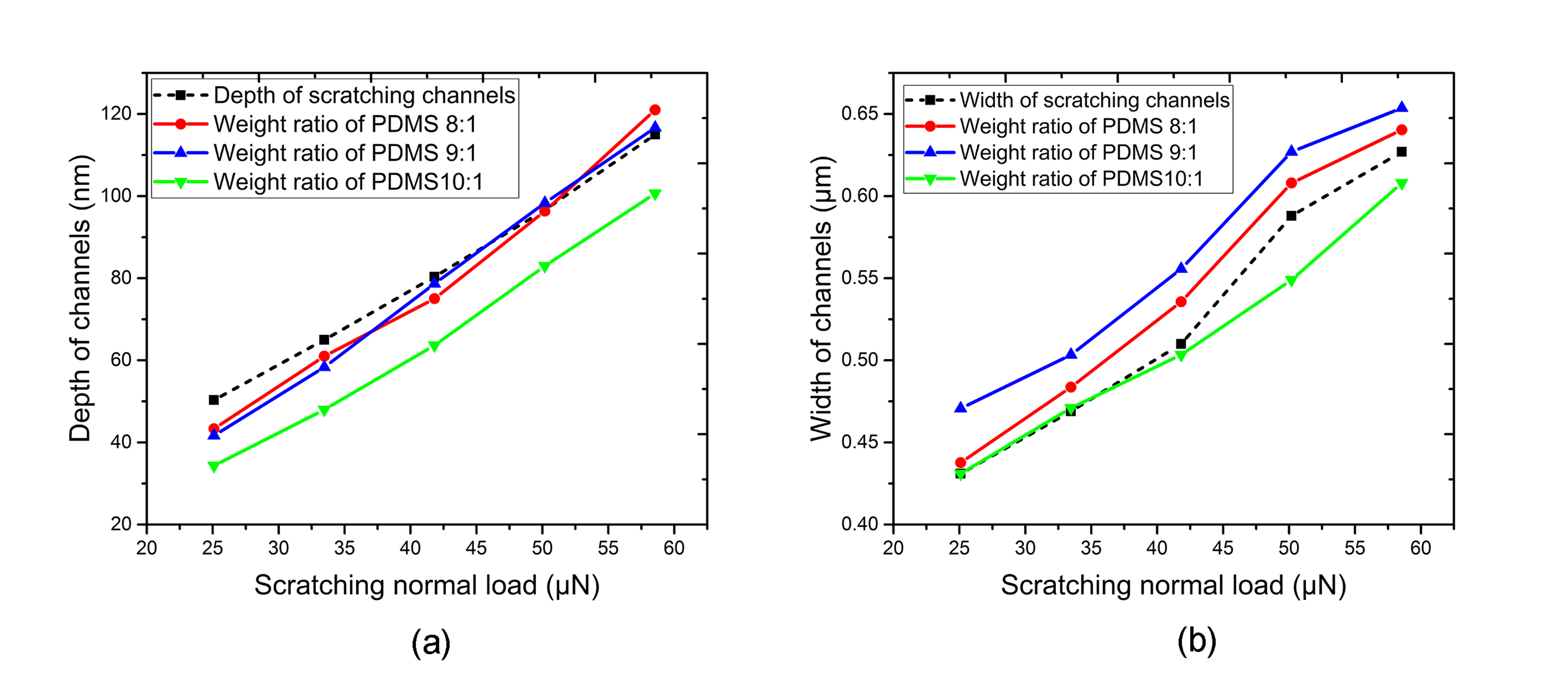

Supplement: Supplementary file 1 — Figure S1. Schematic diagram of homemade alignment system. Figure S2. Schematic illustrations of alignment procedures during bonding process. Figure S3. Relationship between scratching circle diameter and driving voltage. Figure S4. Typical AFM images of the machined nanochannel with different machining parameters. Figure S5. Relationship between wall size and transfer parameters (various weight ratio of PDMS) during first transfer process, where the channel molds were fabricated with single scratching approach: (a) Wall height, (b) Wall width. Figure S6. Typical AFM image (left) and corresponding cross-section (right) of the wall obtained from nanochannel I at a PDMS weight ratio of 5:1 during first transfer. Figure S7. Relationship between wall size and transfer parameters (various weight ratio of PDMS) during first transfer process, where the channel molds were fabricated with a normal load of 17 μN and a frequency of 100 Hz: (a) Wall height, (b) Wall width. Figure S8. Typical AFM image (left) and corresponding cross-section (right) of the wall obtained from nanochannel II at a weight ratio of 5:1 during first transfer. Figure S9. Relationship between nanochannel size and transfer parameters (various weight ratio of PDMS) during second transfer, where the channel molds were fabricated with single scratching approach: (a) Nanochannel depth, (b) Nanochannel width. Figure S10. Typical AFM image (left) and corresponding cross-section (right) of the nanochannel obtained from wall I at a weight ratio of 10:1 during second transfer. Figure S11. Relationship between nanochannel size and transfer parameters (various weight ratio of PDMS) during second transfer, where the channel molds were fabricated with a normal load of 17 μN and a frequency of 100 Hz: (a) Nanochannel depth, (b) Nanochannel width. Figure S12. Typical AFM image (left) and corresponding cross-section (right) of the nanochannel obtained from wall II at a weight ratio of 10:1 during second transfer. (ZIP 16 [file 11671_2019_2962_MOESM1_ESM.zip › Fig. s9.tif]
